# Androgen-Sensitized Apoptosis of HPr-1AR Human Prostate Epithelial Cells

**DOI:** 10.1371/journal.pone.0156145

**Published:** 2016-05-20

**Authors:** Congcong Chen, Jason A. Dienhart, Eric C. Bolton

**Affiliations:** Department of Molecular and Integrative Physiology, University of Illinois at Urbana-Champaign, Urbana, Illinois, United States of America; Northern Institute for Cancer Research, UNITED KINGDOM

## Abstract

Androgen receptor (AR) signaling is crucial to the development and homeostasis of the prostate gland, and its dysregulation mediates common prostate pathologies. The mechanisms whereby AR regulates growth suppression and differentiation of luminal epithelial cells in the prostate gland and proliferation of malignant versions of these cells have been investigated in human and rodent adult prostate. However, the cellular stress response of human prostate epithelial cells is not well understood, though it is central to prostate health and pathology. Here, we report that androgen sensitizes HPr-1AR and RWPE-AR human prostate epithelial cells to cell stress agents and apoptotic cell death. Although 5α-dihydrotestosterone (DHT) treatment alone did not induce cell death, co-treatment of HPr-1AR cells with DHT and an apoptosis inducer, such as staurosporine (STS), TNFt, or hydrogen peroxide, synergistically increased cell death in comparison to treatment with each apoptosis inducer by itself. We found that the synergy between DHT and apoptosis inducer led to activation of the intrinsic/mitochondrial apoptotic pathway, which is supported by robust cleavage activation of caspase-9 and caspase-3. Further, the dramatic depolarization of the mitochondrial membrane potential that we observed upon co-treatment with DHT and STS is consistent with increased mitochondrial outer membrane permeabilization (MOMP) in the pro-apoptotic mechanism. Interestingly, the synergy between DHT and apoptosis inducer was abolished by AR antagonists and inhibitors of transcription and protein synthesis, suggesting that AR mediates pro-apoptotic synergy through transcriptional regulation of MOMP genes. Expression analysis revealed that pro-apoptotic genes (BCL2L11/BIM and AIFM2) were DHT-induced, whereas pro-survival genes (BCL2L1/BCL-XL and MCL1) were DHT-repressed. Hence, we propose that the net effect of these AR-mediated expression changes shifts the balance of BCL2-family proteins, such that androgen signaling sensitizes mitochondria to apoptotic signaling, thus rendering HPr-1AR more vulnerable to cell death signals. Our study offers insight into AR-mediated regulation of prostate epithelial cell death signaling.

## Introduction

Androgen receptor (AR) signaling plays pivotal roles in the development, physiology, and pathology of the prostate gland. Upon binding its endogenous ligands, which include testosterone and 5α-dihydrotestosterone (DHT), a central function of the AR is to regulate gene expression as a transcriptional regulator [1,2]. In the nucleus, ligand-activated ARs associate with specific DNA sequences known as androgen response elements (AREs) and coordinate the recruitment of nuclear co-regulators, chromatin remodeling factors, and the transcriptional machinery, and thus regulate the transcription of target genes [3–9]. AR signaling is a central regulator of normal prostate development, cytodifferentiation, and homeostasis. Further, dysregulation of AR signaling is thought to be responsible for prostate cancer initiation and progression [10].

The oncogenic activity of AR has been intensively studied, mostly in prostate cancer. It is well documented that most prostate cancer cells express AR, and they are somewhat dependent on AR signaling for growth and proliferation [11–21]. In contrast to the oncogenic activity of AR in prostate cancer, AR signaling normally serves to restrain proliferation and stimulate differentiation and survival of luminal prostate epithelial cells in a healthy prostate gland. In humans and rodents, the prostatic epithelium contains basal and luminal layers interspersed with rare neuroendocrine cells. Several groups have shown that intermediate cells and a subset of basal cells express low levels of AR [22–25]. Further, comparison of wild-type littermates to basal-ARKO mice revealed that AR knockout in the basal epithelial cells of the prostate increases the proliferation of these cells, including progenitor/intermediate cells and may decrease the differentiation of these cells to luminal epithelial cells [24]. As intermediate cells differentiate and migrate to the luminal layer, AR expression increases. The abundant expression of AR in luminal epithelial cells is believed to suppress their proliferation and maintain their secretory function [26]. In addition, AR-mediated signaling in the mesenchyme/stroma and a paracrine signaling mechanism may also regulate survival of the luminal epithelial cells in the prostate [27,28].

While AR-regulated cell proliferation has been extensively studied, little is known about the cell stress response and apoptotic functions of AR signaling in prostate epithelial cells, though they are central to growth and homeostasis of the prostate gland. Upon exposure to various intra- or extra- cellular stressors (e.g., inflammatory factors, oxidative stressors, DNA damage agents, toxins, etc.), cells usually initiate multiple pathways to counteract the stimuli and repair the damage. A persistent stress response or irreversible cellular damage activate additional signaling pathways that ultimately lead to programmed cell death [29]. Apoptosis is a highly regulated signaling process that leads to cell death in an energy-dependent manner with characteristic hallmarks [30,31]. Central to apoptotic signaling is the activation of the extrinsic pathway or the intrinsic pathway, which are distinguished by the activation of different caspases. In the extrinsic pathway, death receptors of the tumor necrosis factor receptor superfamily at the plasma membrane sense extracellular death signaling ligands and activate initiator caspase-8 or -10. The active initiator caspases further cleave and activate executor caspases-3, 6, and 7 [32–34]. In the intrinsic pathway, multiple stress signals converge on the mitochondria and cause mitochondrial outer membrane permeabilization (MOMP), which leads to the release of pro-apoptotic factors including cytochrome *c*, apoptosis-inducing factor mitochondrion-associated 1/2 (AIFM1/2), and DIABLO. The released cytochrome *c* is bound by apoptotic peptidase activating factor 1 (APAF1) and assembled into the oligomeric apoptosome, which cleaves and activates initiator caspase-9 and the executor caspases [35–38].

Importantly, the mitochondrial outer membrane and its permeability are crucial mediators of intrinsic and extrinsic apoptotic signaling. Typically, mitochondrial integrity is highly controlled by B-cell CLL/lymphoma 2 (BCL2) family proteins. The pro-apoptotic BCL2-associated X protein (BAX) family proteins (including BAX, BCL2-antagonist/killer 1 (BAK), and BCL2-related ovarian killer (BOK)) mainly function at the mitochondrial membrane. The oligomerization of pro-apoptotic proteins BAX and BAK forms holes at the mitochondrial surface and thus causes the release of pro-apoptotic factors [39]. The pro-survival BCL2 family proteins (including BCL2, BCL2L1/BCL-XL, myeloid cell leukemia 1 (MCL1), etc.) interfere with the pro-apoptotic BCL2 family proteins and therefore maintain mitochondrial membrane integrity [40]. BH-3 only proteins (including BID, BAD, BCL2L11/BIM, NOXA, PUMA, etc.) are also pro-apoptotic, since they interact with pro-survival BCL2 family proteins and prevent them from interfering with BAX and BAK [39]. Indeed, the stoichiometry of pro-survival and pro-apoptotic BCL2 family proteins is a main determinant of whether a cell will live or die [30,40].

AR signaling plays a central role in prostate epithelial homeostasis, which is dependent upon the net balance of cell division and turnover. In a previous study, we defined a growth suppression mechanism whereby AR-mediated down-regulation of cyclin D-CDK4/6 complexes inhibits cell cycle progression of HPr-1AR human prostate epithelial cells [41]. However, AR signaling and the apoptotic stress response of human prostate epithelial cells are not well understood, though they are central signaling pathways that have been implicated in the death of these cells and the regulation of prostate homeostasis and pathology. Importantly, the activation of AR signaling in HPr-1AR cells does not induce apoptosis under optimized culture conditions [41,42], which is consistent with the role that AR signaling serves to maintain homeostasis in the prostate through epithelial cytodifferentiation and growth suppression [24,26,28,43]. To further understand AR signaling in the context of the cell stress response, we hypothesized that AR signaling would promote turnover of stressed and damaged cells due to its homeostatic maintenance function in normal prostate epithelial cells. We investigate this hypothesis in the present study using various cell stress agents.

Here, we demonstrate that AR activation sensitized human prostate epithelial cell lines HPr-1AR and RWPE-AR to apoptotic cell death in response to several cell stress agents, including staurosporine (STS), tumor necrosis factor-alpha and cycloheximide (TNF cycl), and hydrogen peroxide (H_2_O_2_). Interestingly, the mechanism responsible for the androgen-sensitized cell death in HPr-1AR is distinct from the mechanism of AR-mediated growth suppression that we described previously [41]. We found that mitochondria play a central role in androgen-sensitized cell death, and AR-mediated up-regulation of pro-apoptotic genes (BCL2L11/BIM and AIFM2) and down-regulation of pro-survival genes (BCL2L1/BCL-XL and MCL1) may be responsible for the decreases in mitochondrial potential that we measured. Taken together, these data support the hypothesis that AR-mediated gene expression changes shift the balance of BCL2-family proteins, such that androgen signaling sensitizes mitochondria to apoptotic signaling, thus rendering HPr-1AR more vulnerable to cell stress and death signals.

## Materials and Methods

### Cell culture and reagents

Cell lines HPr-1AR and HPr-1 were gifts from Patrick Ming-Tat Ling (University of Hong Kong) [42]. RWPE-AR and RWPE-FG9 (AR-negative control line) were generated by lentiviral transduction of an RWPE-1 clone [44], lacking AR protein expression, as described in Higgins *et al*. and were gifts from Diane Robins (University of Michigan Medical School) [45]. HPr-1AR, HPr-1, RWPE-AR, and RWPE-FG9 cells were grown as described [41,45,46] in keratinocyte serum free medium (17005–042, Invitrogen, Carlsbad, CA) without androgen supplementation and a humidified 5% CO_2_ atmosphere at 37°C. Cells were treated in growth medium with 5 μM CDK4/6 kinase inhibitor, 6-acetyl-8-cyclopentyl-5-methyl-2-[[5-(1-piperazinyl)-2-pyridinyl]amino]pyrido[2,3-d]pyrimidin-7(8H)-one 2-hydroxy-ethanesulfonic acid (1:1), PD0332991, PZ0199, Sigma-Aldrich, St. Louis, MO) or 0–10 nM AR agonist (5α-dihydrotestosterone, DHT) in the presence or absence of AR antagonist, 10 μM 2-hydroxyflutamide (OHF, H4166, Sigma-Aldrich) or 5–10 μM enzalutamide (ENZ, S1250, Selleckchem, Houston, TX), for the indicated duration. To induce apoptotic cell death, cells were further treated with 0.5–2 μM staurosporine (STS, 1285, Tocris, United Kingdom), 40 ng/mL tumor necrosis factor-alpha (TNFα, 210-TA, R&D Systems, Minneapolis, MN) and 2 μg/mL cycloheximide (CHX, C7698, Sigma-Aldrich), 200 μM hydrogen peroxide (H_2_O_2_, H325, Fisher Scientific), or 5 μM (-)-1,1',6,6',7,7'-Hexahydroxy-3,3'-dimethyl-5,5'-bis(1-methylethyl)-[2,2'-binaphthalene]-8,8'-dicarboxaldehyde (AT101, 3367, Tocris Bioscience, Bristol, United Kingdom) for the indicated duration. Cells were co-treated with 20 μg/ml 5,6-dichlororibofuranosylbenzimidazole (DRB, D1916, Sigma-Aldrich) as described [41] to inhibit transcription and 25–50 μg/mL CHX to inhibit protein synthesis.

### Measurement of relative ATP concentrations

The relative ATP levels available for biochemical processes in metabolically active cells were quantified using the CellTiter-Glo Luminescent Cell Viability Assay Kit (G7571, Promega, Madison, WI) as described [41]. Briefly, cells were exposed to 50% CellTiter-Glo Reagent in PBS and luminescence was analyzed using a VICTOR X5 plate reader (2030–0050, PerkinElmer, Waltham, MA).

### Annexin V and propidium iodide (PI) staining

Apoptosis assays were performed using Alexa Fluor 488- or Pacific Blue-conjugated Annexin V/Dead Cell Apoptosis Kit (A13201 or A35122, Molecular Probes, Crand Island, NY), as instructed by the manufacturer, and quantified using an LSR II flow cytometer (Becton Dickinson Biosciences, Franklin Lakes, NJ) and FCS Express 4 Flow software (De Novo Software, Glendale, CA). Briefly, cells were collected, washed with cold PBS, and resuspended in 100–150 μL of binding buffer. Cells were stained with 5–7.5 μL of annexin V and 1–1.5 μL of 100 μg/mL PI at room temperature for 15 min and then diluted with 250–400 μL of binding buffer. The intensities of fluorescent annexin V and PI stained cells were quantified by flow cytometry at 450 nm, 530 nm and 695 nm, respectively. The percentages of cells undergoing apoptosis were determined by dual color analysis, which allowed us to distinguish three subsets of cells: viable live cells (annexin V-negative and PI-negative), early apoptotic cells (annexin V-positive and PI-negative), and late apoptotic or necrotic cells (annexin V-positive and PI-positive) [47,48].

### Immunoblot analysis

Immunoblots were performed to determine the relative levels of cleaved and activated caspase-8, caspase-9, and caspase-3 as well as the relative expression of BCL2L11/BIM, BCL2L1/BCL-XL, and MCL1 at various time points after the indicated treatment. Cells were washed with PBS and harvested in 200–600 μL of RIPA buffer (20 mM Tris-HCl (pH 7.5), 150 mM NaCl, 1 mM Na_2_EDTA, 1 mM EGTA, 1% Triton X-100, 1% sodium deoxycholate, 2.5 mM sodium pyrophosphate, 1 mM β-glycerophosphate, 1 mM NaF and protease inhibitors). Total cell lysates containing equal amounts of protein were subjected to 4–20% mini-TGX gel (456–1096, Bio-Rad, Hercules, CA) electrophoresis for 35 min at 200 V and then transferred to PVDF membranes for 2 or more hours at 50 V. Non-specific binding was blocked by incubation of the membranes in a solution containing 5% non-fat milk in TBST buffer (20 mM Tris-HCl (pH 7.5), 150 mM NaCl and 0.1% Tween-20). Blots were incubated with rabbit antibodies raised against GAPDH, caspase-3, cleaved caspase-3, caspase-8, caspase-9, BCL2L11/BIM, BCL2L1/BCL-XL, and MCL1 (2118, 9662, 9664, 9496, 9502, 2933, 2764, and 5453, Cell Signaling, Danvers, MA) overnight, followed by incubation with anti-rabbit IgG, horseradish peroxidase (HRP)-linked antibody (7074 Cell Signaling). The blots were visualized using Pierce ECL Western Blotting Substrates (32106/34080/34096, Thermo, Rockford, IL) and HyBlot CL Autoradiography Film (E3018, Denville Scientific, Metuchen, NJ). Films were scanned and Image J software was used to quantify protein expression relative to GAPDH.

### Mitochondrial membrane potential assay

The mitochondrial membrane potential of treated HPr-1AR cells was detected using the MitoProbe JC-1 Assay Kit (M34152, Molecular Probes, Crand Island, NY), as instructed by the manufacturer, and quantified by flow cytometry. Cells were harvested and resuspended in 1 mL warm PBS. JC-1 dye was added to a final concentration of 5 μM. In addition, the positive control group was exposed to 100 μM carbonyl cyanide 3-chlorophenylhydrazone (CCCP), which is a proton ionophore that has been shown to uncouple oxidative phosphorylation in mitochondria [49,50]. Cells were then incubated in a humidified 5% CO_2_ atmosphere at 37°C for 20 minutes. After two washes with PBS at 37°C, cells were resuspended in 1 mL PBS, and the intensities of fluorescent JC-1 stained cells were quantified by flow cytometry at 530 nm and 585 nm.

### RNA isolation, reverse transcription, and real-time quantitative polymerase chain reaction (QPCR)

Total RNA was extracted from the cells using Qiagen RNeasy Mini Kit (74106, Qiagen) with on-column DNase treatment (79254, Qiagen). Random-primed cDNA was prepared from 2 μg of total RNA using the ProtoScript First Strand cDNA kit (E6300L, New England Biolabs, Ipswich, MA) and diluted 5-fold in water. One μl of diluted cDNA was used as template for QPCR using StepOnePlus Real-Time PCR System (Applied Biosystems, Grand Island, NY). Primers were designed using Primer3 (http://frodo.wi.mit.edu) and those that efficiently amplified [51] single products of the expected size were used for QPCR. Primers for QPCR amplicons are in Supporting Information (S1 Table). The QPCR was achieved using the following method: a denaturation and polymerase activation step at 94°C for 1 min and then 40 cycle consisting of 94°C for 10 s, 57°C for 10 s, and 72°C for 20 s. Data were analyzed using the comparative threshold cycle (Ct) method [52] and multiple control genes, including ADAM15, PYGO2, TBP and GAPDH, which are not regulated by androgen or AR. Following normalization to control gene cDNA levels, which is reflected in the ΔCt values, the relative quantification (RQ) of the fold change for each treatment compared to reference control was determined using the following equation: RQ = 2^(nd mu^ / 2^(/ 2m reference)^. The mean of the log_2_ RQ and standard error of the mean (SEM) were plotted.

### Statistical analysis

Unless otherwise specified, the significance differences between two treatment groups were determined by Student’s t-test, whereas the differences among multiple groups were determined by analysis of variance (ANOVA) using a randomized complete block design, followed by Tukey's honest significant difference test. To improve the validity of ANOVA, non-normal or heteroscedastic data were normalized using the corresponding basal level or transformed using variance stabilization transformation methods (e.g., arcsine transformation and Box-Cox power transformation) before ANOVA [53]. A significance level of 0.05 was applied during data analysis, and different significance levels have been indicated using different lowercase letters. In addition, the ANOVA data from the analysis of the viable live cells (annexin V-negative and PI-negative, gray bars) that were quantified by flow cytometry are provided in Supporting Information (S2 Table). We have indicated the 95% confidence intervals of the main and interaction effects.

## Results

### Androgen and staurosporine synergize to decrease the relative ATP concentration of HPr-1AR cells

**Fig 1:**
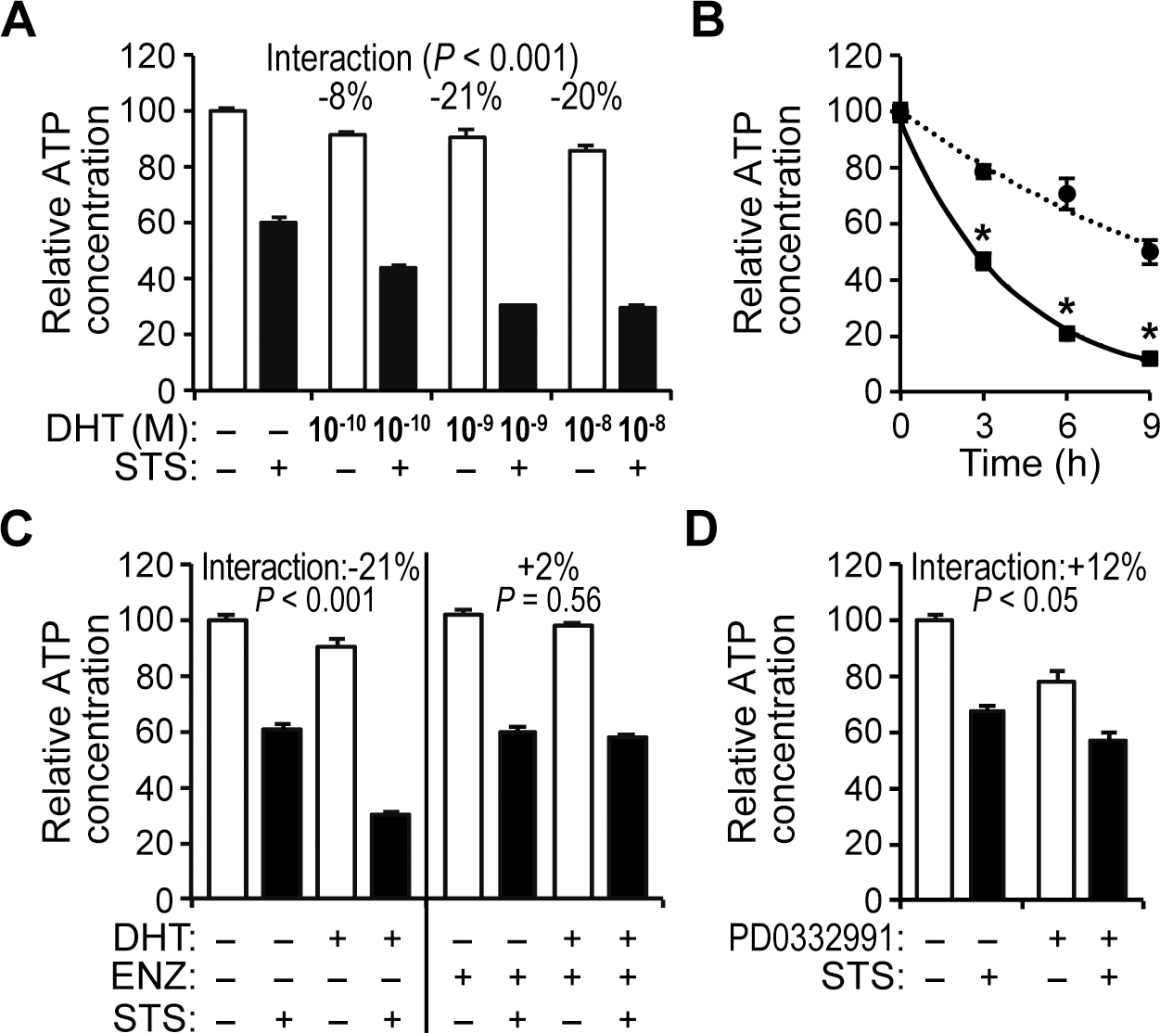
Androgen and staurosporine synergize to decrease the relative ATP concentration in HPr-1AR cells. (A) HPr-1AR cells were treated with a range of DHT concentrations or vehicle control for 18 hours and then co-treated with 1 μM STS or vehicle control for 6 hours. Relative ATP concentrations available for biochemical processes in metabolically active cells were quantified using a luciferase-based bioassay for relative ATP levels in cultured cells. In comparison to vehicle control, STS and to a lesser extent DHT significantly decrease the relative ATP concentration of HPr-1AR cells at 24 hours. In addition, ANOVA revealed significant interaction between 1 μM STS and 0.1–10 nM DHT, which is visually evident from the unparallel trends of the white bars and black bars in the plot. Estimates of the interaction effect and corresponding p-values are indicated. Negative interaction terms indicate synergy whereas positive values indicate antagonism between DHT and STS. (B) Cells were treated with 10 nM DHT or vehicle control for 10 hours and then co-treated with 0.5 μM STS for 0, 3, 6, or 9 hours (h). In comparison to control-treated HPr-1AR cells (circles), DHT-treated HPr-1AR cells (squares) had 40%, 72%, and 76% reductions in ATP levels after 3, 6, and 9 hours of STS co-treatment, respectively. For time course analysis, significance differences between androgen treatment and vehicle control were determined at each time point using Student’s t-test and adjusted using the Bonferroni method, * *P* < 0.05. (C) Cells were treated with 1 nM DHT or vehicle control in the absence or presence of 5 μM enzalutamide (ENZ) for 18 hours and then co-treated with 1 μM STS or vehicle control for 6 hours. AR antagonist, ENZ significantly suppresses the synergistic interaction between DHT and STS. (D) Cells were treated with 5 STS. (suppresses the es between andof CDK4/6 kinase activity, for 18 hours to mimic the inhibitory effect of DHT on HPr-1AR cell cycle progression and growth, and then these cells were co-treated with 1 μM STS or vehicle control for 6 hours. The positive interaction term indicates that the synergy between DHT and STS on ATP depletion is not dependent on growth suppression and suggests an antagonistic effect between STS and PD0332991. Data represent the mean ± SEM (n ta4).

**Fig 2:**
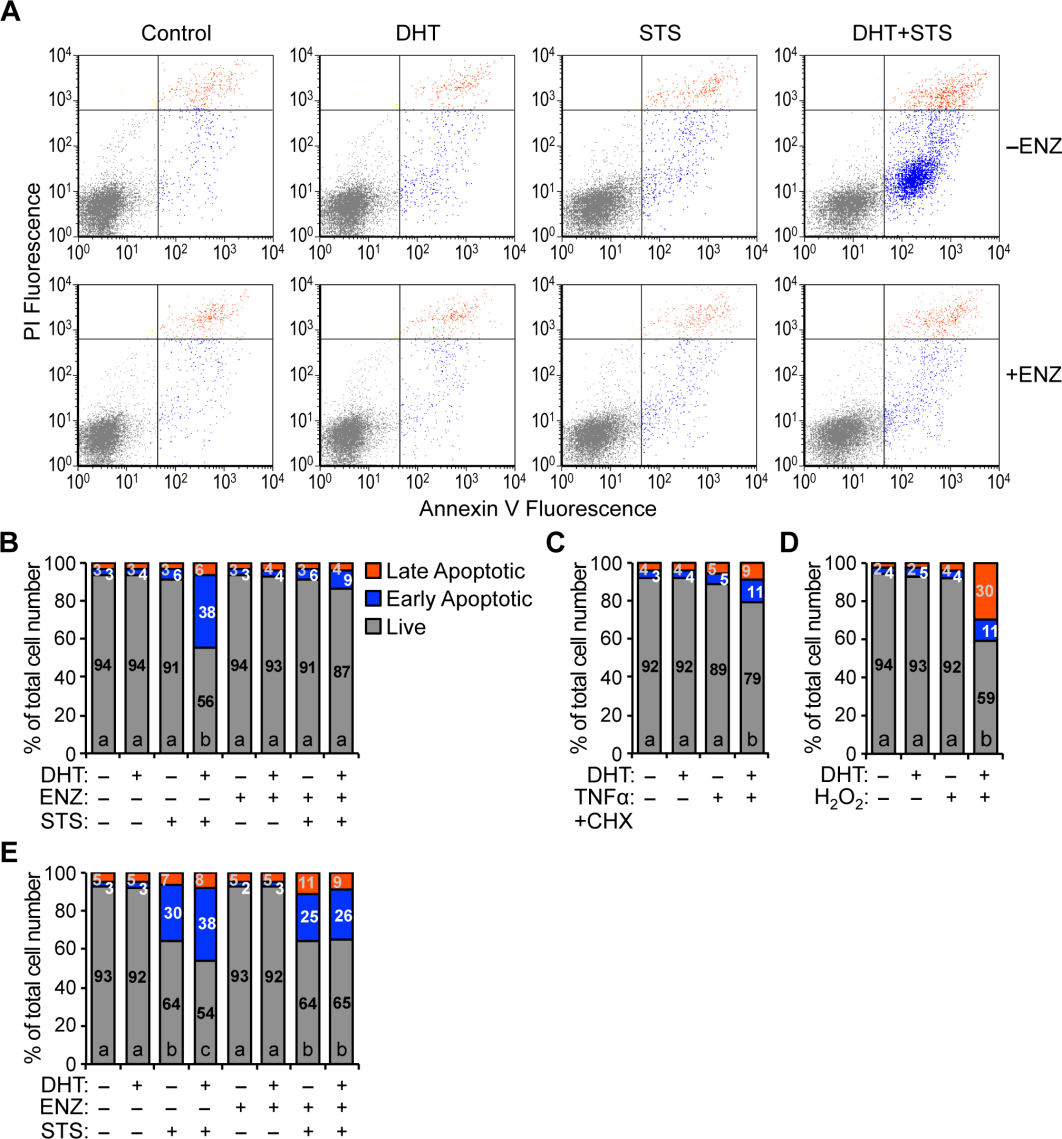
Androgen sensitizes HPr-1AR and RWPE-AR to apoptotic cell death. (A) HPr-1AR cells were treated with 1 nM DHT or vehicle control in the absence or presence of 5 μM ENZ for 19 hours and then co-treated with 1 μM STS or vehicle control for 5 hours. Cells were harvested, stained with annexin V and PI, and the fluorescence intensities of annexin V and PI stained cells were quantified by flow cytometry. Viable live cells (annexin V-negative and PI-negative, gray dots), early apoptotic cells (annexin V-positive and PI-negative, blue dots), and late apoptotic cells (annexin V-positive and PI-positive, orange dots) are indicated. (B) Quantification of the fraction of viable live (gray bar with black number), early apoptotic (blue bar with white number), and late apoptotic cells (orange bar with gray number) is shown from the dot plots in Fig 2A. DHT treatment alone does not trigger cell death in HPr-1AR. However, DHT sensitizes HPr-1AR to STS-induced apoptosis. In addition, AR antagonist, ENZ, suppresses the synergistic interaction between DHT and STS, which significantly increases the live cell proportion. (C) HPr-1AR cells were treated with 1 nM DHT or vehicle control for 12 hours and then co-treated with apoptosis inducer, TNFoptos, or vehicle control for 11 hours. The fluorescence intensities of annexin V and PI stained cells were then quantified by flow cytometry. DHT sensitizes HPr-1AR cells to apoptotic death induced by TNF sens. (D) HPr-1AR cells were treated with 1 nM DHT or vehicle control for 20 hours and then co-treated with apoptosis inducer, H_2_O_2_, or vehicle control for 24 hours. The fluorescence intensities of annexin V and PI stained cells were then quantified by flow cytometry. DHT sensitizes HPr-1AR cells to apoptotic death induced by H_2_O_2_. (E) RWPE-AR cells were treated with 1 nM DHT or vehicle control in the absence or presence of 5 μM ENZ for 30 hours and then co-treated with 1 μM STS or vehicle control for 10 hours. The fluorescence intensities of annexin V and PI stained cells were then quantified by flow cytometry. DHT treatment alone does not induce cell death in RWPE-AR. However, DHT sensitizes RWPE-AR to STS-induced apoptosis. Further, ENZ co-treatment completely suppresses the synergistic interaction between DHT and STS, fully rescuing the live cell proportion of RWPE-AR. Data represent the mean (n yn3). Comparisons between multiple treatment groups were performed using three- or two-way ANOVA followed by Tukey's honest significant difference test (S2 Table).

The mechanism whereby AR influences the proliferation and survival of prostate epithelial cells has been investigated. Our group and Ling *et al*. have previously reported that androgens inhibit the proliferation of HPr-1AR, a human prostate epithelial cell line stably expressing wild-type AR (S1 Fig), without affecting cell survival under optimized culture conditions [41,42]. However, the cellular stress response of human prostate epithelial cells is not well understood. To investigate whether androgen signaling may modulate the stress response of prostate epithelial cells, we treated HPr-1AR cells with 0–10 nM 5α-dihydrotestosterone (DHT) for 18 hours followed by 1 μM staurosporine (STS), a widely-used cell stress agent and apoptosis inducer [54], or DMSO vehicle control and then measured relative ATP concentrations at 24 hours. A two-way analysis of variance (ANOVA) model was applied to quantify the effects of DHT and STS alone as well as any interaction between DHT and STS. In dose-response experiments (Fig 1A), 0.1 to 10 nM DHT treatments modestly decreased the relative ATP concentration (8% to 14%) at 24 hours due to DHT-induced growth suppression of HPr-1AR cells as previously described [41]. Meanwhile, STS treatment by itself decreased the relative ATP levels by 40%. Interestingly, DHT and STS co-treatment significantly decreased ATP concentrations 8% to 21% beyond the additive effects of DHT and STS treatments (p<0.001), indicating that DHT and STS synergize to reduce the relative ATP levels in HPr-1AR. In time-course experiments of HPr-1AR cells pre-treated with DHT compared to vehicle as a control, the sensitizing effect of DHT on STS-induced ATP depletion was evident at 3, 6, and 9 hours, decreasing ATP levels by 40–76% (Fig 1B). In addition, the synergistic interaction between DHT and STS was suppressed by AR antagonists, enzalutamide (ENZ) (Fig 1C) and 2-hydroxyflutamide (OHF) (S2 Fig). Hence, the androgen-sensitized depletion of ATP concentration in HPr-1AR is mediated by AR and may lead to a synergistic decrease of viability.

Further, to assess whether the synergistic depletion of ATP concentration by DHT and STS is due to a prolonged G_1_ interval in the cell cycle and growth suppression [41], we treated HPr-1AR cells with 5 μM PD0332991, a selective inhibitor of CDK4/6 kinase activity, to mimic the inhibitory effect of DHT on HPr-1AR cell cycle progression and growth. PD0332991 by itself decreased the relative ATP concentration by 20% due to growth suppression, and STS by itself decreased ATP concentration and, possibly, viability by 35% (Fig 1D). However, the co-treatment group showed no synergistic effect. Rather, the interaction between STS and PD0332991 in an additive model was +12% (Fig 1D), suggesting an antagonistic effect between STS and PD0332991. Nonetheless, the synergy of DHT and STS on ATP depletion is not dependent on growth suppression. Hence, the mechanism whereby AR sensitizes HPr-1AR cells to STS-induced ATP depletion and, possibly, cell death is distinct from AR-mediated growth suppression.

### Androgen sensitizes HPr-1AR and RWPE-AR to apoptotic cell death

To determine whether the synergistic decrease of ATP concentration in HPr-1AR cells co-treated with DHT and STS was due to an increase in programmed cell death, we performed annexin V and propidium iodide (PI) staining, which was quantified using flow cytometry. Healthy live cells stain poorly with annexin V and PI. Early apoptotic cells that expose more phosphatidylserine at the surface of their intact plasma membrane are labeled by annexin V but not PI, whereas the nuclei of late apoptotic cells with compromised membrane integrity are labeled by annexin V and PI. In the absence of STS treatment, DHT by itself did not induce HPr-1AR cell death (Fig 2A and 2B, S2 Table), which is consistent with a previous study using terminal deoxynucleotidyl transferase dUTP nick end labeling (TUNEL) assay [42]. STS by itself increased the apoptotic population, albeit not significantly. However, DHT and STS co-treatment synergistically increased the proportion of dead cells by 35% compared to STS treatment alone. Further, ENZ suppressed the synergistic interaction between DHT and STS, which is evident from the rescue of the live cell population. Consistent with the synergistic effect of DHT and STS being AR-mediated, control experiments using the AR-negative parental cell line HPr-1 (S1 Fig) revealed a lack of synergy between DHT and STS (S3 Fig, S2 Table).

To test whether the DHT-sensitized cell death is limited to STS-induced apoptosis, we treated HPr-1AR with other cell stress agents, including tumor necrosis factor-alpha and cycloheximide (TNFclCHX), hydrogen peroxide (H_2_O_2_), and AT101, which can induce apoptosis through different mechanisms that are distinct from STS [55–59]. Although DHT by itself did not induce cell death, co-treatment of any of these cell stress agents with DHT significantly increased the proportions of early and late apoptotic cells. DHT co-treatment potentiated TNF the proportions of -treby 10% (Fig 2C, S2 Table), H_2_O_2_-induced cell death by 33% (Fig 2D, S2 Table), and AT101-induced apoptosis by 15% (data not shown). Thus, AR signaling in HPr-1AR is pro-apoptotic in the sense that it sensitizes HPr-1AR cells to apoptotic death in response to cell stress agents.

In addition, we investigated the extent to which androgen sensitizes RWPE-AR cells to programmed cell death. RWPE-AR is a clone of the RWPE-1 human prostate epithelial cell line [44] that stably expresses wild-type AR (S1 Fig). DHT by itself did not induce RWPE-AR cell death (Fig 2E, S2 Table), which is consistent with our findings in HPr-1AR (Fig 2A and 2B). STS by itself significantly increased the proportion of apoptotic RWPE-AR cells (Fig 2E). DHT and STS co-treatment synergistically increased the proportion of dead cells by 10% compared to STS treatment alone. Further, ENZ abolished the synergistic interaction between DHT and STS, which is demonstrated by the complete rescue of the live cell population. Consistent with the synergy of DHT and STS being AR-mediated, additional control experiments using AR-negative RWPE-FG9 control cells (S1 Fig) showed an absence of synergy between DHT and STS (S3 Fig, S2 Table). Indeed, the HPr-1AR and RWPE-AR data reveal that physiologic levels of DHT sensitize benign, AR-expressing human prostate epithelial cells to programmed cell death.

**Fig 3:**
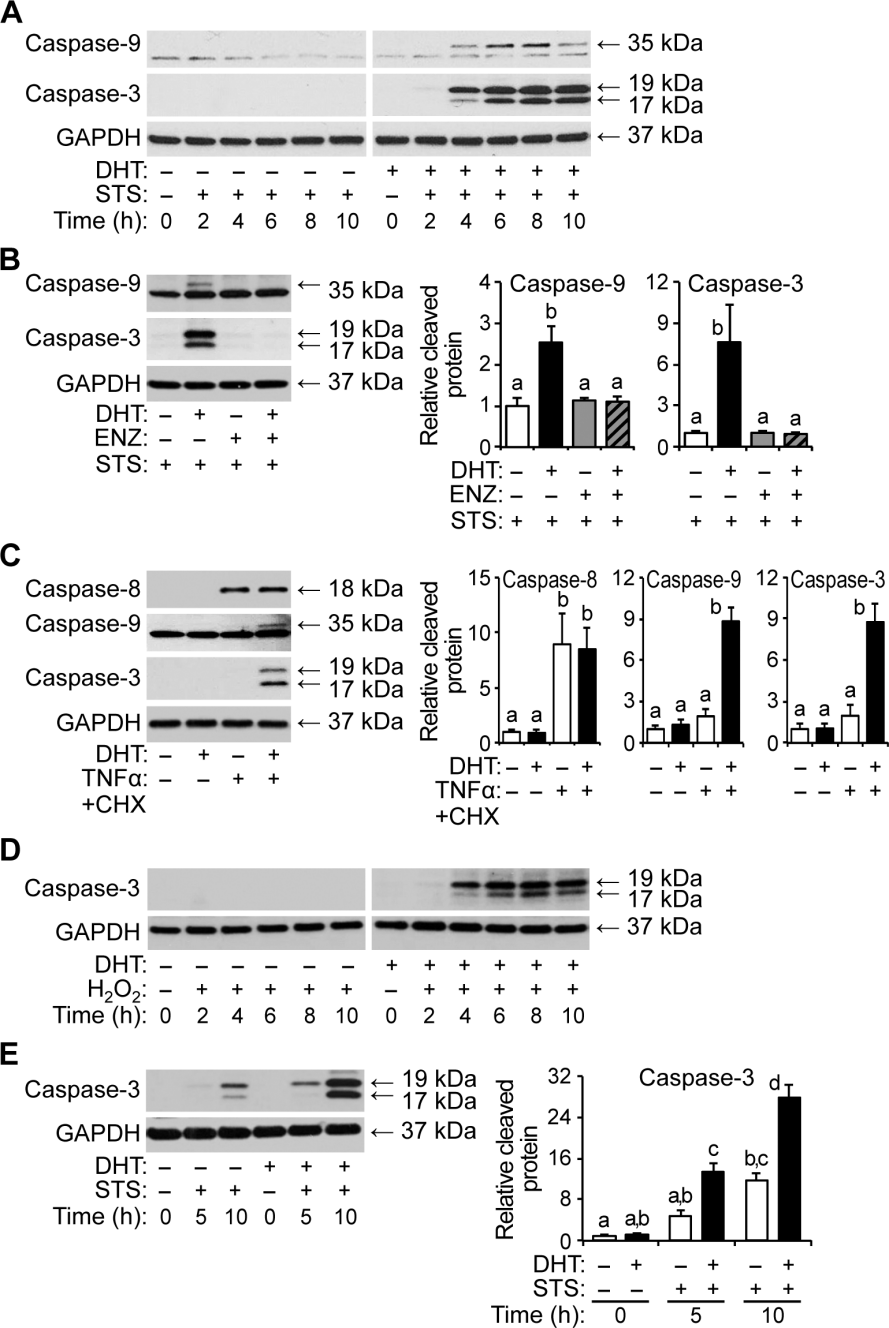
Androgen-sensitized apoptosis of HPr-1AR cells involves caspase activation. (A) HPr-1AR cells were treated with 10 nM DHT or vehicle control for 18 hours and co-treated with 1 μM STS for 0 to 10 hours. Immunoblot analysis was performed using antibodies that detect the cleaved and active forms of caspase-9 (35 kDa) and caspase-3 (19 and 17 kDa). HPr-1AR cells pretreated with DHT show rapid activation of caspase-9 and caspase-3 upon STS co-treatment, whereas DHT or STS treatment alone show little or no caspase activation. (B) Cells were treated with 1 nM DHT or vehicle control in the absence or presence of 5–10 μM ENZ for 18 hours and then co-treated with 1 μM STS or vehicle control for 6 hours. The DHT-induced cleavage of caspase-9 and caspase-3 in STS-treated HPr-1AR cells is completely suppressed by AR antagonist, ENZ. (C) Cells were treated with 1–10 nM DHT or vehicle control for 18 hours and then co-treated with TNFated or vehicle control for 10 hours. Immunoblot analysis was performed using an additional antibody to detect the cleaved and active form of caspase-8 (18 kDa), an initiator caspase that is activated in response to extrinsic apoptotic stimuli, such as TNFe. DHT and TNF and synergistically enhance cleavage of caspase-9 and caspase-3, whereas DHT or TNF or treatment alone shows no significant activation of caspase-9 or caspase-3. The arrows and corresponding molecular weights indicate the different caspase forms. (D) HPr-1AR cells were treated with 1 nM DHT or vehicle control for 24 hours and co-treated with 200 μM H_2_O_2_ for 0 to 10 hours. HPr-1AR cells pretreated with DHT show rapid activation of caspase-3 upon H_2_O_2_ co-treatment, whereas DHT or H_2_O_2_ alone show little or no caspase-3 activation. (E) RWPE-AR cells were treated with 1 nM DHT or vehicle control for 38 hours and then co-treated with 1 μM STS or vehicle control for 5 to 10 hours. RWPE-AR cells pretreated with DHT show rapid and robust activation of caspase-3 upon STS co-treatment (11-fold at 5 hours and 23-fold at 10 hours) compared to RWPE-AR cells pretreated with vehicle as a control. Immunoblot results were quantified and represented as the mean ± SEM (n ± S. Comparisons between different treatments were performed using two-way ANOVA followed by Tukey's honest significant difference test.

To confirm that androgen sensitizes HPr-1AR to apoptosis rather than other types of cell death, we examined the proteolytic cleavage of caspases, which become activated and serve as a hallmark of the later stages of apoptosis. In accordance with the annexin V and PI staining results, DHT treatment by itself did not increase the cleavage of initiator caspase-9 or executioner caspase-3 (Fig 3A). However in combination with STS, DHT robustly increased cleaved caspase-9 and caspase-3 levels. In time course experiments, DHT and STS co-treatment rapidly enhanced the levels of cleaved caspase-9 and caspase-3 (within 4 hours), whereas STS treatment by itself had little or no effect on the cleavage of these caspases (Fig 3A). Importantly, the DHT- and STS-enhanced cleavage of caspase-9 and caspase-3 was completely blocked by AR antagonist, ENZ (Fig 3B). In addition, activation of extrinsic apoptotic signaling using TNFα+CHX lead to cleavage and activation of initiator caspase-8 (Fig 3C). Although synergistic activation of caspase-8 was not detected in cells co-treated with DHT and TNFα+CHX, DHT synergized with TNFα+CHX to robustly enhance cleavage of caspase-9 and caspase-3 in HPr-1AR cells (Fig 3C). In additional time course experiments, DHT and H_2_O_2_ co-treatment rapidly increased the levels of cleaved caspase-3 (within 4 hours), whereas H_2_O_2_ treatment by itself had little or no effect on caspase-3 cleavage (Fig 3D). Consistent with the HPr-1AR data, DHT and STS co-treatment robustly enhanced the levels of cleaved caspase-3 in RWPE-AR (within 5 hours), whereas STS treatment by itself modestly increased caspase-3 cleavage (within 10 hours) (Fig 3E). Taken together, the annexin V and PI staining data and the caspase activation data demonstrate that AR-mediated androgen signaling sensitizes HPr-1AR and RWPE-AR cells to apoptosis through caspase activation, which may involve the intrinsic mitochondrial pathway.

### Androgen and staurosporine synergize to induce mitochondrial depolarization in HPr-1AR

Mitochondria play crucial roles in cell survival and death decisions. Mitochondrial outer membrane permeabilization (MOMP) and the release of cytochrome *c* and other pro-apoptotic molecules are early signaling events that lead to activation of the intrinsic apoptotic pathway and they may also be activated by the extrinsic apoptotic pathway. To determine whether androgen sensitizes HPr-1AR to undergo apoptosis by inducing MOMP, we performed JC-1 staining followed by flow cytometry analysis of treated HPr-1AR cells. The dual-fluorescent dye, JC-1, accumulates in mitochondria and serves as a sensor of mitochondrial membrane potential, which is an important parameter of mitochondrial health that declines rapidly during MOMP.

**Fig 4:**
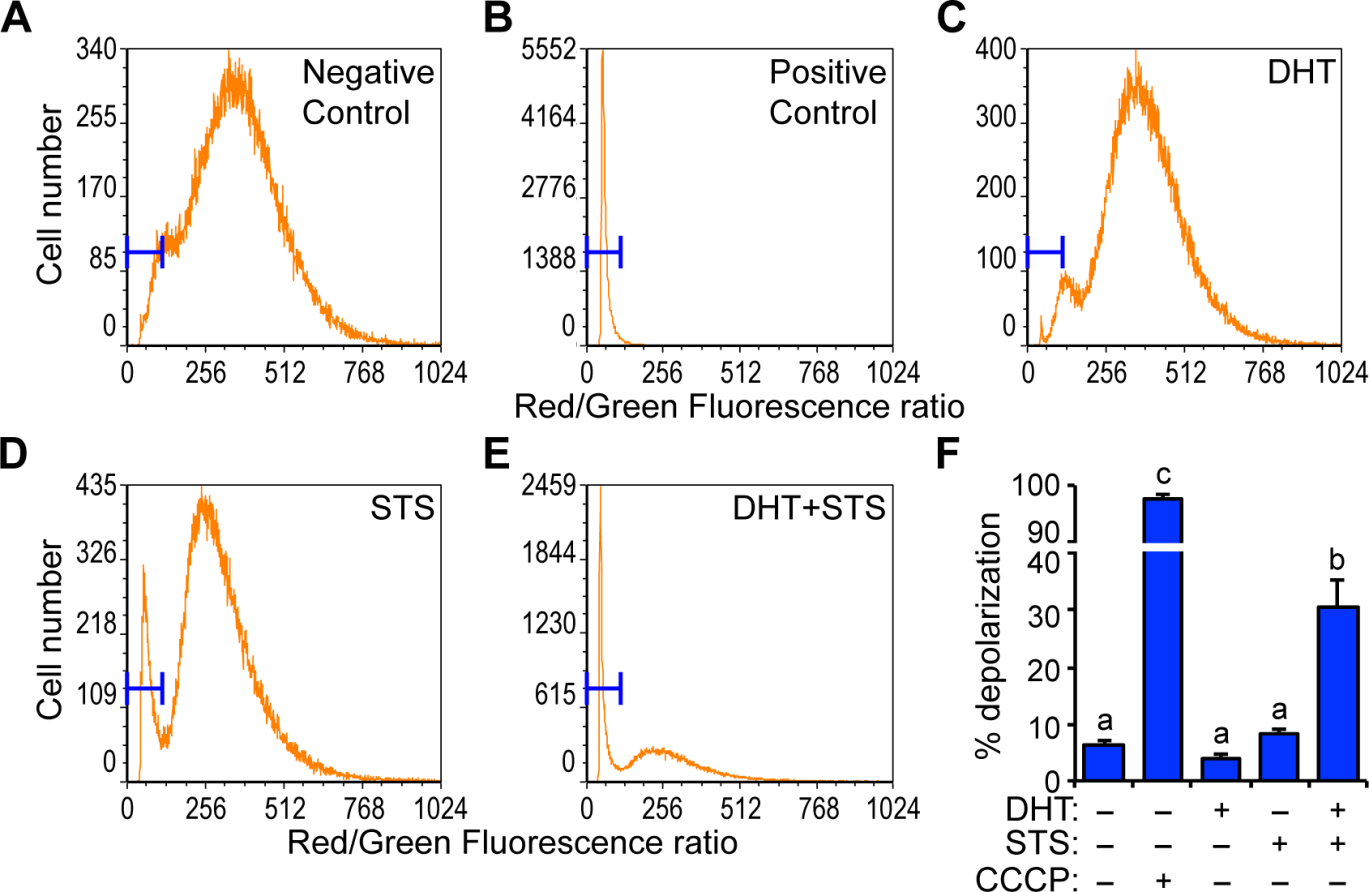
Androgen and staurosporine synergize to induce mitochondrial depolarization in HPr-1AR. (A-E) HPr-1AR cells were treated with 10 nM DHT or vehicle control for 20 hours and then co-treated with 2 μM STS or vehicle control for 4.5 hours. Cells treated with vehicle control were used as the negative control. In addition, 2 μM CCCP was added to an aliquot of the negative control cells to depolarize their mitochondria and generate the positive control. Cells were stained with JC-1 dye and analyzed using flow cytometry. For each treatment, the ratios of red to green fluorescent intensities are displayed in histograms. Blue brackets at the left of each panel specify the population of cells with depolarized mitochondria. Normal cells (A) have a wide and bell-shaped distribution of JC-1 stained mitochondria, whereas cells with depolarized mitochondria have a sharp and left-shifted distribution (B). (F) Quantification of the depolarized cell proportion for each treatment revealed that DHT or STS alone do not significantly increase mitochondrial depolarization. However, co-treatment with DHT and STS significantly increases the depolarized population by 4-fold or more. Data represent the mean ± SEM (n = 4). Comparisons between different treatments were performed using two-way ANOVA followed by Tukey’s honest significant difference test.

Normal cells with intact mitochondria exhibit strong red and green fluorescence, display various red to green ratios, and thus form a wide bell-shaped distribution in a histogram plot (Fig 4A). In contrast, cells with depolarized mitochondria due to treatment with 100 μM carbonyl cyanide 3-chlorophenylhydrazone (CCCP), which is a proton ionophore that has been shown to uncouple oxidative phosphorylation in mitochondria [49,50], exhibit low red fluorescence, display uniformly low red to green ratios, and form a left-shifted spike in a histogram plot (Fig 4B). DHT treatment did not increase mitochondrial depolarization (Fig 4C) and STS treatment by itself modestly increases mitochondrial depolarization, as evidenced by a sub-peak close to zero in the histogram (Fig 4D). Remarkably, DHT and STS co-treatment substantially increased the proportion of cells with depolarized mitochondria by 4-fold or more (Fig 4F), which resulted in a left-shifted spike in the histogram (Fig 4E), approximating the shape of the positive control sample (compare Fig 4B and 4E). Since the mitochondrial depolarization is usually a direct outcome of MOMP, these results indicate that AR-sensitized apoptosis in HPr-1AR involves increased mitochondrial permeability.

### Transcription and protein synthesis are necessary for androgen-sensitized apoptosis of HPr-1AR

**Fig 5:**
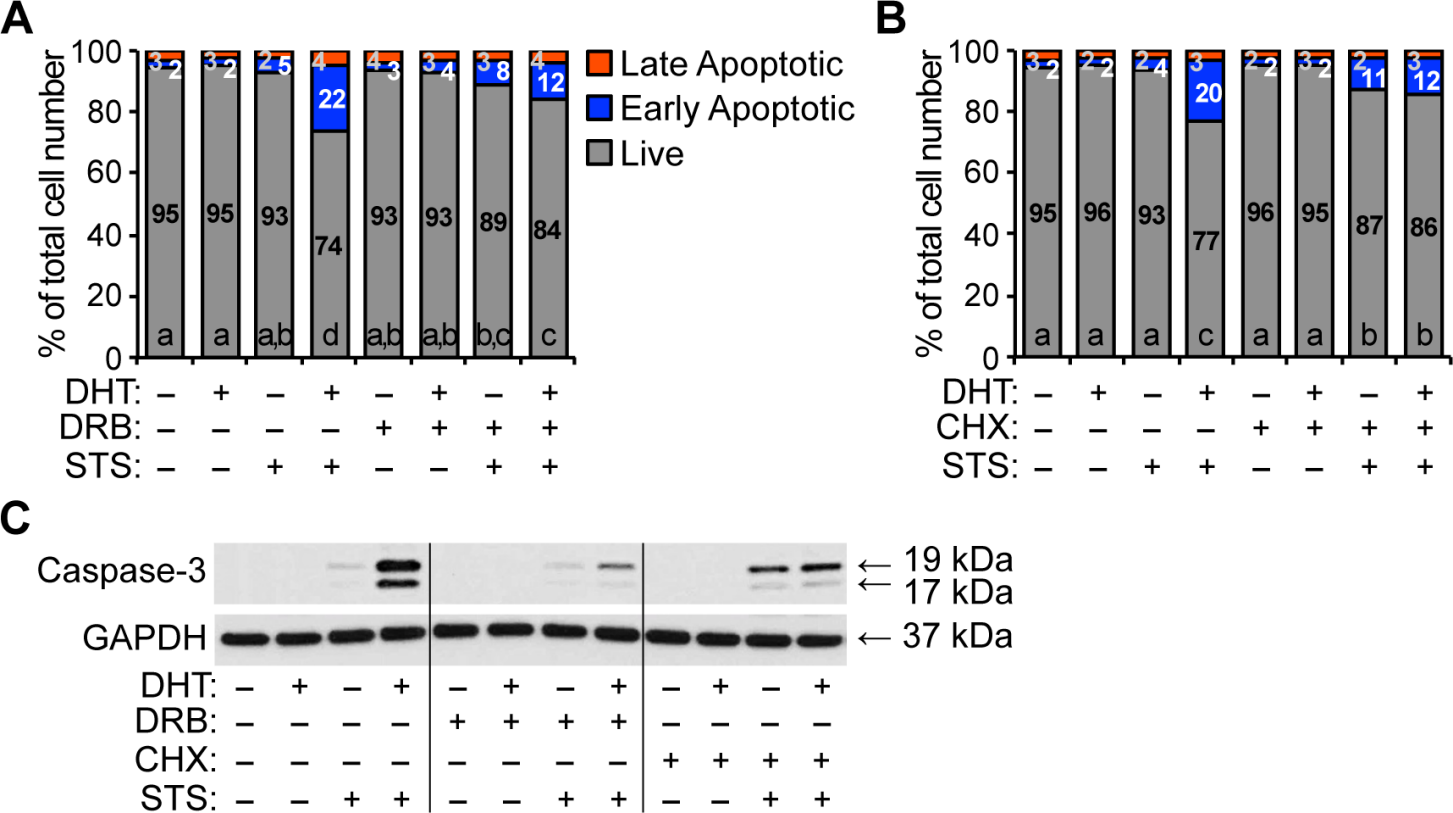
Transcription and protein synthesis are necessary for androgen-sensitized apoptosis of HPr-1AR. (A) Cells were treated with 1 nM DHT or vehicle control in the absence or presence of transcription inhibitor, 20 in the5,6-dichlororibofuranosylbenzimidazole (DRB), for 16 hours, and then these cells were co-treated with 1 μM STS or vehicle control for 4 hours to induce apoptosis. Cells were harvested, stained with annexin V and PI, and the intensities of annexin V and PI stained cells were quantified by flow cytometry. DRB treatment significantly suppressed the androgen-sensitized apoptosis of HPr-1AR. (B) HPr-1AR cells were treated with 1 nM DHT or vehicle control in the absence or presence of protein synthesis inhibitor, 25 μg/mL CHX, for 16 hours, and then these cells were co-treated with 1 μM STS or vehicle control for 4 hours to induce apoptosis. Cells were harvested, stained with annexin V and PI, and analyzed by flow cytometry. CHX co-treatment completely suppressed the androgen-sensitized apoptosis of HPr-1AR. Data represent the mean (n = 3). Comparisons between multiple treatment groups were performed using three-way ANOVA followed by Tukey's honest significant difference test (S2 Table). (C) Immunoblot analysis of cell lysates reveals that DHT-induced caspase-3 cleavage in STS-treated HPr-1AR cells is significantly suppressed by the inhibition of transcription (DRB) and protein synthesis (CHX).

The androgen-sensitized apoptosis in HPr-1AR was suppressed by AR antagonists (Figs 2A and 2B and 3B), implicating AR in this process. Previous studies have reported that AR displays pro-apoptotic function; although the proposed mechanisms differ between these studies, a common assertion is that the pro-apoptotic function of AR is independent of the transcriptional regulation function of AR [60–64]. To investigate whether the AR-mediated apoptotic signaling is dependent on transcription, we blocked transcription in HPr-1AR cells using 5,6-dichlororibofuranosylbenzimidazole (DRB). HPr-1AR cells were treated with 1 nM DHT or vehicle control in the absence or presence of 20 μg/mL DRB for 16 hours, and then co-treated with 1 μM STS or vehicle control for 4 hours to induce apoptosis. To assess cell death, we performed annexin V and PI staining, which was quantified using flow cytometry. DRB treatment significantly suppressed the androgen-sensitized apoptosis of HPr-1AR (Fig 5A, S2 Table). In addition, we blocked protein synthesis in HPr-1AR using 25 μg/mL CHX in place of DRB and assessed cell death by quantifying annexin V and PI staining. Similar to DRB, CHX treatment significantly suppressed the androgen-sensitized apoptosis of HPr-1AR (Fig 5B, S2 Table). Immunoblot analysis of caspase-3 cleavage revealed that CHX by itself modestly increased caspase-3 cleavage in the presence of STS. Further, inhibition of transcription by DRB or protein synthesis by CHX, robustly suppressed the synergy between DHT and STS in HPr-1AR. (Fig 5C). Taken together, these data demonstrate that transcription and protein synthesis are necessary for androgen-sensitized apoptosis in HPr-1AR.

### AR-mediated transcriptional regulation of apoptotic genes in HPr-1AR

**Fig 6:**
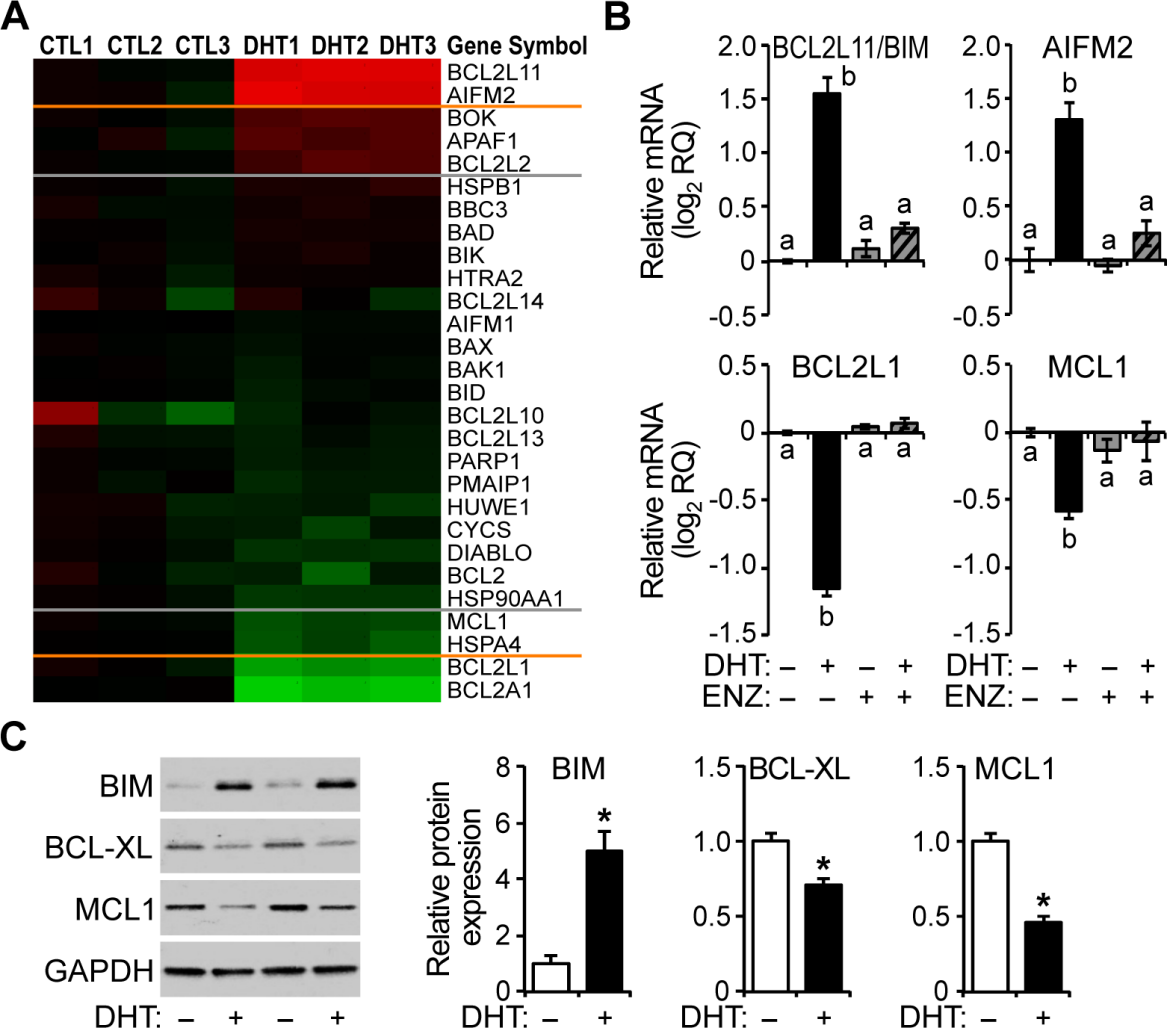
AR-mediated transcriptional regulation of apoptotic genes in HPr-1AR. (A) Cells were treated with 10 nM DHT or vehicle control for 24 hours. Total RNA was isolated, cDNA was synthesized by reverse transcription, and the relative mRNA levels of apoptosis-related genes were quantified by QPCR analysis. The colorimetric representation shows genes, indicated by HUGO Gene Nomenclature Committee gene symbols, whose transcripts were induced (red), unresponsive (black), and repressed (green) by androgen. The color intensity reflects the relative fold change (brightest red = 4-fold increase and brightest green = 4-fold decrease) in transcript level for DHT- versus vehicle control (CTL)-treated cells (n = 3). The 2-fold cutoff boundaries (orange lines) and 1.5-fold cutoff boundaries (gray lines) were determined from the mean change in expression level for DHT- versus CTL-treated cells. (B) Cells were treated with 1 nM DHT or vehicle control in the absence or presence of 5 μM ENZ for 24 hours. QPCR analysis demonstrates that the pro-apoptotic genes (BCL2L11 and AIFM2) are DHT-induced, whereas the pro-survival genes (BCL2L1 and MCL1) are DHT-repressed. Further, the AR antagonist, ENZ, completely suppressed the androgen-responsive mRNA changes. Data represent the mean ± SEM (n = 3) in log_2_ scale. Comparisons between different treatments were performed using two-way ANOVA followed by Tukey’s honest significant difference test. (C) HPr-1AR cells were treated with 10 nM DHT for 24 hours. Immunoblot analysis of BCL2 family gene products demonstrates that the expression of pro-apoptotic BCL2L11/BIM is androgen-induced, whereas pro-survival proteins, BCL2L1/BCL-XL and MCL1, are androgen-repressed. Representative immunoblots (left panel) and the quantified results (right panels) are shown. Data represent the mean ± SEM (n = 4). Comparisons between DHT- versus vehicle-treated cells were made using Student’s t-test, * *P* < 0.05.

To identify genes responsible for androgen-sensitized MOMP in HPr-1AR and to further investigate whether androgen-sensitized apoptosis of these cells is dependent on the transcriptional regulation function of AR, we interrogated the expression of genes implicated in the mitochondrial apoptotic pathway using QPCR. In HPr-1AR, the mRNA levels of several apoptotic genes changed significantly with DHT treatment (Fig 6A). Specifically, several BCL2 family genes were androgen-responsive, including the DHT-induced pro-apoptotic genes (BCL2L11/BIM and BOK) and the DHT-repressed pro-survival genes (BCL2A1, BCL2L1/BCL-XL, and MCL1) (Fig 6A and 6B). In addition, transcripts for the AIFM2 gene, which codes for a pro-apoptotic protein that is released from the mitochondria into to the cytoplasm upon MOMP, and the APAF1 gene, which codes for an apoptosis initiator protein that binds cytochrome *c* and forms the oligomeric apoptosome, were DHT-induced. The androgenic regulation of pro-apoptotic gene (BCL2L11/BIM and AIFM2) transcripts and pro-survival gene (BCL2L1/BCL-XL and MCL1) transcripts was blocked by co-treatment with AR antagonist ENZ, implicating AR in their transcriptional regulation (Fig 6B). Further, immunoblot analysis confirmed that the expression of pro-apoptotic BCL2L11/BIM, was up-regulated, and pro-survival factors, BCL2L1/BCL-XL and MCL1, were down-regulated (Fig 6C). Taken together, these results identify androgen-responsive genes (BCL2L11/BIM, AIFM2, BCL2L1/BCL-XL, and MCL1), which are known to express apoptotic regulators, as candidate targets for AR-mediated transcriptional regulation.

## Discussion

**Fig 7:**
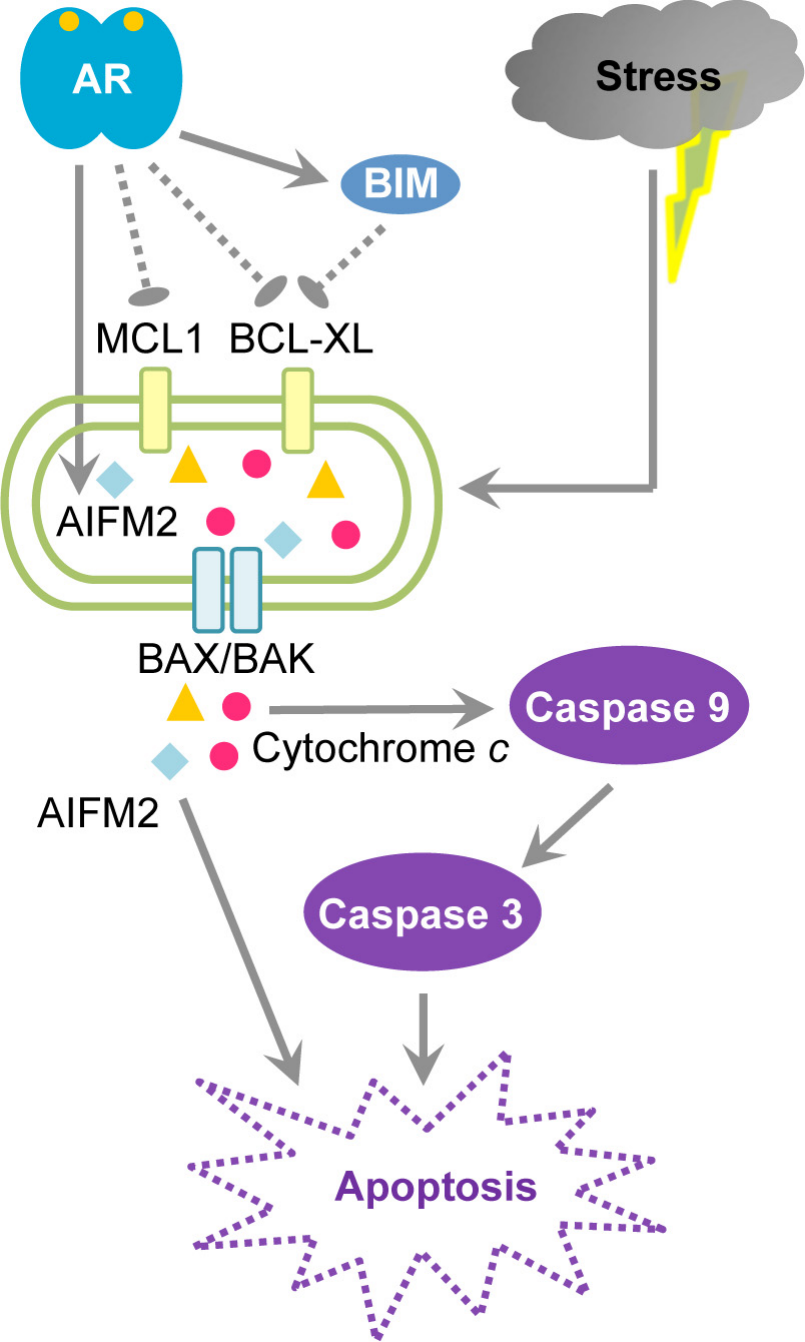
A model for androgen-sensitized apoptosis of HPr-1AR. Activation of AR by agonists, such as DHT, enhances AR nuclear accumulation and transcriptional regulation of target genes, including genes involved in the regulation of apoptosis. We propose that AR exerts pro-apoptotic roles by modestly up-regulating the expression of pro-apoptotic genes (BCL2L11/BIM and AIFM2) and down-regulating the expressions of pro-survival genes (BCL2L1/BCL-XL and MCL1). The modest changes in the expression of the BCL2 family proteins may explain why DHT treatment alone does not induce apoptosis in HPr-1AR cells. However, the multi-target regulation of BCL2 family proteins by AR signaling may sufficiently stress the mitochondria towards a pro-apoptotic state (i.e., leaky outer membrane) and decrease the threshold for other apoptotic stimuli to further enhance permeabilization of the mitochondrial outer membrane above the necessary threshold for initiator and executioner caspase activation (caspase-9 and caspase-3, respectively) and apoptotic disassembly of the cells.

We investigated the functional role of AR in the apoptotic stress response of human prostate epithelial cells. We determined that AR signaling alone does not induce HPr-1AR and RWPE-AR cell death, rather it exerts pro-apoptotic activity that sensitizes these human prostate epithelial cells to apoptotic cell death in response to various cell stress agents. Our results indicate that AR-mediated regulation of mitochondrial membrane integrity plays a central role in this process. BCL2 family proteins function as “molecular switches” that fine-tune mitochondrial membrane permeability. Our study shows that DHT up-regulates the expression of pro-apoptotic genes (including BCL2L11/BIM and AIFM2) and down-regulates the expressions of pro-survival genes (including BCL2L1/BCL-XL and MCL1) indicating that AR signaling may exert its pro-apoptotic activity on mitochondrial membrane permeability by altering the expression of BCL2 family genes. Notably, the regulation of BCL2 family gene expression is modest, which may explain why DHT treatment alone does not induce apoptosis in HPr-1AR. However, the multi-target regulation of BCL2 family proteins by AR signaling may sufficiently stress the mitochondria toward a pro-apoptotic state (i.e., leaky outer membrane) and decrease the threshold for other cell stress agents to deliver the deathblow through enhanced permeabilization of the mitochondrial outer membrane above the threshold for caspase activation and apoptosis (Fig 7).

### AR action in non-malignant prostate epithelial cells

AR signaling plays crucial roles in prostate homeostasis, which involves balancing cell division, differentiation, and death. Studies *in vivo* using mice lacking AR in cytokeratin-5 expressing cells of the prostate basal epithelial layer have demonstrated that AR signaling suppresses basal epithelial and progenitor cell proliferation and drives basal epithelial cells toward a more differentiated state [24,28]. Further, AR normally promotes cytodifferentiation and suppresses proliferation of intermediate epithelial cells [24] and maintains the secretory function of differentiated luminal epithelial cells [28]. In addition, androgen activation of AR expressed in the basal, intermediate, and luminal epithelial cells from rodents and humans invariably suppresses cell cycle progression and proliferation of these cells [24,26,41,42].

Previously, we described a mechanism of androgen-suppressed proliferation of HPr-1AR cells that is AR-mediated and involves down-regulation of cyclin D1/2-CDK4/6 complexes and up-regulation of cell cycle inhibitor, CDKN1A/p21 [41]. In comparison to quiescent cells, proliferating cells have been suggested to be more susceptible to apoptosis. The activation of cyclins and CDKs are positively correlated with the onset of apoptosis [65–68], whereas, the up-regulation of cell cycle inhibitors has been suggested to decrease susceptibility to apoptosis [67,69,70]. Based on these previous studies, the anti-proliferative effect of androgen in HPr-1AR and the expression changes of the cyclins, CDKs, and CDKN1A in response to androgen were expected to suppress apoptosis in HPr-1AR. However, we found that activation of AR signaling sensitizes HPr-1AR and RWPE-AR cells to apoptosis in response to cell stress agents. Furthermore, our results using PD0332991, which inhibits CDK4/6-mediated cell cycle progression, demonstrates that the anti-proliferative function of AR signaling is distinct from its pro-apoptotic function in HPr-1AR (Fig 1D).

### Apoptotic functions of AR signaling

While AR-mediated growth suppression has been well-studied, little is known about the apoptotic functions of AR in the prostate epithelium. Previous studies have revealed that androgen treatment does not induce apoptosis of human or rodent prostate epithelial cells [26,41,42]. As the experiments described in these studies were conducted under optimized culture conditions and in the absence of cellular stress, they primarily focused on defining the role of AR signaling in growth suppression and differentiation rather than its role in the cell stress response. To further investigate the apoptotic functions of AR, we challenged HPr-1AR cells with several different cell stress agents. Surprisingly, HPr-1AR cells pre-treated with DHT displayed hypersensitivity and synergy to several apoptosis inducers, including STS, TNFα+CHX, H_2_O_2_, and AT101 (Figs 2–3). Importantly, these cell stress agents are known to induce cell death through different mechanisms. STS, H_2_O_2_, and AT101 stimulate the intrinsic apoptotic pathway by non-selective inhibition of protein kinases [54], oxidative stress and damage [57–59], and suppression of pro-survival BCL2 family genes [55], respectively; whereas, TNFspectively; whereas5" \o "Balakrishnan, 2009 #117" Year>1986</TNF family death receptors located at the cell surface [56]. Hence, the androgen-sensitized apoptosis in HPr-1AR appears to be a common response to cell stress agents and further implicates crosstalk between AR and apoptotic signaling pathways, rather than simple cytotoxic effects.

In addition to their central role in intrinsic apoptotic signaling pathways, mitochondria are also downstream targets of extrinsic signaling pathways, and therefore serve as apoptotic signaling hubs. Our finding that androgen sensitizes HPr-1AR cells to intrinsic and extrinsic apoptosis led us to question whether the putative crosstalk between AR and apoptotic signaling pathways involves mitochondria. The JC-1 staining profile showed a significant trend whereby DHT and STS synergistically increase mitochondrial membrane permeability (Fig 4), suggesting that DHT renders mitochondria more vulnerable to intrinsic apoptotic stimuli and MOMP. Meanwhile, the activation of caspase-9, a hallmark of intrinsic apoptotic signaling, was detected after co-treatment with DHT and TNFas de (Fig 3). Activation of intrinsic signaling following activation of extrinsic signaling indicates that the androgen-sensitized apoptosis of HPr-1AR is correlated with increased mitochondrial outer membrane permeability. This conclusion is also consistent with our gene expression analysis. Three pro-survival genes (BCL2A1, BCL2L1/BCL-XL, and MCL1) were DHT-repressed and four pro-apoptotic genes (BCL2L11/BIM, AIFM2, BOK, and APAF1) were DHT-induced (Fig 6). The net expression changes in the BCL2 family gene products are likely sufficient to increase the probability of hole formation at the mitochondrial surface and therefore lower the threshold for MOMP. The moderate regulation of these BCL2 family genes may be the reason that DHT treatment alone fails to induce MOMP and apoptosis in HPr-1AR.

AR promoted cell death has been described in several prostate cancer cell lines. Androgen-induced apoptosis, characterized by DHT induced-cell cycle arrest and DNA fragmentation, has been reported for PC-3 cells that stably overexpressed AR [71]. However, more recent studies have shown that androgen inhibits the proliferation of AR-expressing PC-3 cells without inducing cell death [72]. In LNCaP-104-R1, which are castration-resistant prostate cancer cells that express mutant AR, AR signaling has been suggested to promote ultraviolet or STS-induced apoptosis via BAX protein translocation from the cytoplasm to mitochondria [73]. Additional studies elucidated that the pro-apoptotic function of the AR is primarily mediated by the proteasome, which generates N-terminal fragments of the AR that may decrease the basal expression of CDKN1A (p21) through a transcription-independent mechanism. Further, blockade of AR degradation or overexpression of BCL2 was found to suppress the pro-apoptotic function of AR in LNCaP-104-R1 [63,64]. Such a mechanism is not consistent with the androgen-sensitized apoptosis of HPr-1AR because we have shown previously that CDKN1A is a DHT-induced gene in HPr-1AR [41]. In AR-negative DU145 cells, co-expression of RB and AR reportedly induces intrinsic apoptosis that is dependent on pro-apoptotic proteins, BAX and BAK. However, the apoptotic effect is dependent on RB-mediated transactivation of AR rather than AR ligand binding [74]. In polyglutamine (polyQ)-expanded AR-induced cell death, the polyQ fragments, generated by caspase-3 cleavage, have been suggested to mediate their cytotoxic effect by BAX-dependent intrinsic pathway activation [60,62]. Nonetheless, nearly all of these apoptotic effects of AR have been suggested to be mediated by “non-genomic” AR function outside of the nucleus [75]. Therefore, the mechanism whereby androgen sensitizes HPr-1AR cells to death differs from the previous reports in two significant aspects. First, the pro-apoptotic AR function depends on agonist binding, as AR antagonists abolished the synergistic interaction between DHT and STS (Figs 1–3). Second, AR exerts pro-apoptotic function through transcriptional regulation, since transcriptional inhibition robustly suppressed the synergy between DHT and STS (Fig 5).

### Insights for prostate cancer

In contrast with the anti-proliferative and pro-apoptotic roles of AR in non-malignant prostate epithelial cells, most prostate cancer cells express AR and are somewhat dependent on AR signaling for growth and proliferation [11–21,75]. In these cells, pro-survival signaling by AR has been suggested to prevent apoptosis through multiple mechanisms [69,76–81]. Therefore, androgen deprivation therapy (ADT) has been applied as a first-line treatment for metastatic prostate cancer and the beneficial effects are evidenced by rapid and dramatic tumor regression [82–84]. However, nearly all prostate cancers treated with ADT become resistant to its effects over a period of months to years, as a subset of the malignant cells eventually adapts to survive the ADT-induced environment and emerges as castration-resistant prostate cancer (CRPC).

Our results in HPr-1AR suggest a mechanism that may, in part, explain the development of CRPC. From tumor histology studies, it is well known that prostate cancer cells most closely resemble secretory prostate epithelial cells, however recent biomarker and genomic studies have revealed that prostate cancers are commonly multifocal and consist of heterogeneous cell populations [85,86]. Recent studies have demonstrated that both basal and luminal epithelial cells can initiate prostate cancer in mouse models [87–90]. Importantly, AR expression in benign basal/progenitor cells is known to be low or undetectable, which is consistent with a lack of AR-mediated cytodifferentiation and growth suppression [24] and resistance to castration-induced cell death. AR signaling may retain similar functional roles in prostate cancer stem progenitor (S/P) cells. As such, ADT may stimulate the proliferation and survival of these cells. Therefore, it is reasonable to speculate that distinct cell populations arise in prostate cancer, including a population that has become androgen-stimulated for growth and other populations that are androgen-independent or androgen-suppressed for growth. While ADT may induce cell cycle inhibition and even death in the androgen-stimulated population, the drop of serum androgen levels may also loosen the brake imposed on the androgen-suppressed population, providing a selective advantage for these cells to survive, proliferate, and support tumor recurrence. In support of this, Zhifang *et al*. recently reported that AR activation suppressed the proliferation and tumorigenesis of S/P cells isolated from LNCaP prostate cancer cell line [91]. Although AR-mediated growth suppression of S/P cells remains to be confirmed *in vivo*, these results taken together with our results in HPr-1AR and RWPE-AR are consistent with cell-specific roles of AR signaling in the prostate. Hence, the development of therapies that selectively inhibit the proliferative and anti-apoptotic AR activities while preserving the anti-proliferative and pro-apoptotic AR activities may provide a beneficial outcome compared to systemic inhibition of AR signaling.

### AR-mediated transcriptional regulation of apoptosis-related genes

The AR-sensitized apoptosis in HPr-1AR was blocked using AR antagonists and inhibitors of transcription and protein synthesis (Fig 5), indicating that AR-regulated gene expression is necessary for the synergy of DHT and the cell stress agents. We interrogated apoptosis-related genes using QPCR to identify androgen-responsive genes that may play a role in AR-sensitized apoptosis. We found several androgen-responsive genes are putative AR-regulated genes, whereas the vast majority of the apoptosis-related genes were unresponsive to androgen. Of the apoptosis-related genes with the greatest response to androgen, the pro-apoptotic genes (BCL2L11/BIM, AIFM2, BOK, and APAF1) are DHT-induced, and the pro-survival genes (BCL2A1, BCL2L1/BCL-XL, and MCL1) are DHT-repressed. Interestingly, most of these androgen-responsive genes are BCL2 family genes, which are known to regulate mitochondrial membrane permeability, and the expression changes of these genes in response to androgen are consistent with pro-apoptotic AR activity in HPr-1AR.

To further investigate whether these androgen-responsive genes may be regulated by AR, we searched for putative AR-occupied sites near these genes using the Hormone Receptor Target Binding Loci Database (HRTBLDb) [92] and ENCODE Consortium data [93]. The Massie *et al*. data in HRTBLDb using R1881-treated LNCaP cells reveal an AR-occupied region 81 kb downstream of the AIFM2 TSS as a putative site of AR regulation [94]. Since the closely related glucocorticoid receptor (GR) binds *in vitro* with similar affinity as AR to consensus sequences in the elementary half-sites GGTACAnnnTGTTCT [3,46], we also searched GR-occupied regions near these genes. The ENCODE Consortium data for dexamethasone-treated A549 cells identify multiple GR-occupied regions near all seven of the putative AR-regulated apoptosis-related genes [93]. Hence, the AR-regulated AIFM2, APAF1, and BCL2 family genes are putative AR targets. Importantly, future experiments designed to examine the transcriptional rates of these genes, the stability of their transcripts, and AR occupancy at these genes are needed to demonstrate AR-mediated transcriptional regulation of these genes.

### Conclusions

In conclusion, we report that AR exerts pro-apoptotic function in HPr-1AR and RWPE-AR human prostate epithelial cells. Although androgen alone does not induce cell death, it sensitizes HPr-1AR and RWPE-AR to cell stress agents that induce apoptosis. We also found that the pro-apoptotic function of AR requires transcription. We further propose that AR-mediated transcriptional activation of pro-apoptotic genes (including BCL2L11/BIM and AIFM2) and transcriptional repression of pro-survival genes (including BCL2L1/BCL-XL and MCL1) lowers the threshold necessary to achieve mitochondrial outer membrane permeabilization, thus rendering HPr-1AR more vulnerable to cell stress and death signals. Taken together, our study offers insights into the apoptotic function and mechanism of AR signaling in the androgen-responsive gene network of prostate epithelial cells. Future studies are needed to investigate whether pro-apoptotic signaling by AR is common in other cellular contexts and *in vivo*.

## Supporting Information

S1 Fig AR protein expression in HPr-1AR and RWPE-AR cell lines. Immunoblots show robust AR protein expression in HPr-1AR and RWPE-AR lysates compared to HPr-1 and RWPE-FG9 lysates. AR protein expression is nearly 3-fold higher in HPr-1AR compared to RWPE-AR.
(TIF)

S2 Fig AR antagonist, 2-hydroxyflutamide, suppresses synergy between androgen and staurosporine to rescue HPr-1AR cell viability. Cells were treated with 1 nM DHT or vehicle control and 10 μM 2-hydroxyflutamide (OHF) for 18 hours and then co-treated with 1 μM STS or vehicle control for 6 hours. AR antagonist, OHF, significantly suppresses the synergistic interaction between DHT and STS. Data represent the mean ± SEM (n = 4).
(TIF)

S3 Fig Absence of synergy between androgen and staurosporine in HPr-1 and RWPE-FG9 cell lines, which lack AR protein expression. (A) HPr-1 cells were treated with 1 nM DHT or vehicle control 21 hours and then co-treated with 0.5–1 μM STS or vehicle control for 4 hours. Cells were harvested, stained with annexin V and PI, and the fluorescence intensities of annexin V and PI stained cells were quantified by flow cytometry. Quantification of the fraction of viable live (gray bar with black number), early apoptotic (blue bar with white number), and late apoptotic cells (orange bar with gray number) is shown. DHT treatment alone does not trigger cell death in HPr-1. Further, DHT does not sensitize HPr-1 to STS-induced apoptosis. (B) RWPE-FG9 cells were treated with 1–10 nM DHT or vehicle control for 29 hours and then co-treated with 1 μM STS or vehicle control for 10 hours. The fluorescence intensities of annexin V and PI stained cells were then quantified by flow cytometry. DHT treatment alone does not induce cell death in RWPE-FG9. Further, DHT does not sensitize RWPE-FG9 to STS-induced apoptosis. Data represent the mean (n = 3). Comparisons between multiple treatment groups were performed using two-way ANOVA followed by Tukey's honest significant difference test (S2 Table).
(TIF)

S1 Table Primers for QPCR amplicons. (TIF)

S2 Table ANOVA data from live cell populations quantified by flow cytometry. (TIF)
