# Correction: Androgen-Sensitized Apoptosis of HPr-1AR Human Prostate Epithelial Cells

**DOI:** 10.1371/journal.pone.0213800

**Published:** 2019-03-13

**Authors:** Congcong Chen, Jason A. Dienhart, Eric C. Bolton

There is an error in the fifth sentence of the Abstract. The correct sentence is: Although 5α-dihydrotestosterone (DHT) treatment alone did not induce cell death, co-treatment of HPr-1AR cells with DHT and an apoptosis inducer, such as staurosporine (STS), TNFα, or hydrogen peroxide, synergistically increased cell death in comparison to treatment with each apoptosis inducer by itself.

There are a number of errors in the third paragraph of the Introduction. The complete, correct paragraph is:

While AR-regulated cell proliferation has been extensively studied, little is known about the cell stress response and apoptotic functions of AR signaling in prostate epithelial cells, though they are central to growth and homeostasis of the prostate gland. Upon exposure to various intra- or extra- cellular stressors (e.g., inflammatory factors, oxidative stressors, DNA damage agents, toxins, etc.), cells usually initiate multiple pathways to counteract the stimuli and repair the damage. A persistent stress response or irreversible cellular damage activates additional signaling pathways that ultimately lead to programmed cell death [29]. Apoptosis is a highly regulated signaling process that leads to cell death in an energy-dependent manner with characteristic hallmarks [30,31]. Central to apoptotic signaling is the activation of the extrinsic pathway or the intrinsic pathway, which are distinguished by the activation of different caspases. In the extrinsic pathway, death receptors of the tumor necrosis factor receptor superfamily at the plasma membrane sense extracellular death signaling ligands and activate initiator caspase-8 or -10. The active initiator caspases further cleave and activate executioner caspases-3, -6, and -7 [32–34]. In the intrinsic pathway, multiple stress signals converge on the mitochondria and cause mitochondrial outer membrane permeabilization (MOMP), which leads to the release of pro-apoptotic factors including cytochrome c, apoptosis-inducing factor mitochondrion-associated 1/2 (AIFM1/2), and DIABLO. The released cytochrome c is bound by apoptotic peptidase activating factor 1 (APAF1) and assembled into the oligomeric apoptosome, which cleaves and activates initiator caspase-9 and the executioner caspases [35–38].

There is an error in the first sentence of the last paragraph of the Introduction. The correct sentence is:

Here, we demonstrate that AR activation sensitized human prostate epithelial cell lines HPr-1AR and RWPE-AR to apoptotic cell death in response to several cell stress agents, including staurosporine (STS), tumor necrosis factor-alpha and cycloheximide (TNFα+CHX), and hydrogen peroxide (H_2_O_2_).

In the “RNA isolation, reverse transcription, and real-time quantitative polymerase chain reaction (QPCR)” subsection of the Materials and Methods, there is an error in the penultimate sentence of the paragraph. The correct sentence is: Following normalization to control gene cDNA levels, which is reflected in the ΔCt values, the relative quantification (RQ) of the fold change for each treatment compared to reference control was determined using the following equation: RQ = 2^(-ΔCt)^ / 2^(-ΔCt reference)^.

There are a number of errors in the caption for Fig 1, “Androgen and staurosporine synergize to decrease the relative ATP concentration in HPr-1AR cells.” Please see the complete, correct Fig 1 caption here.

**Fig 1:**
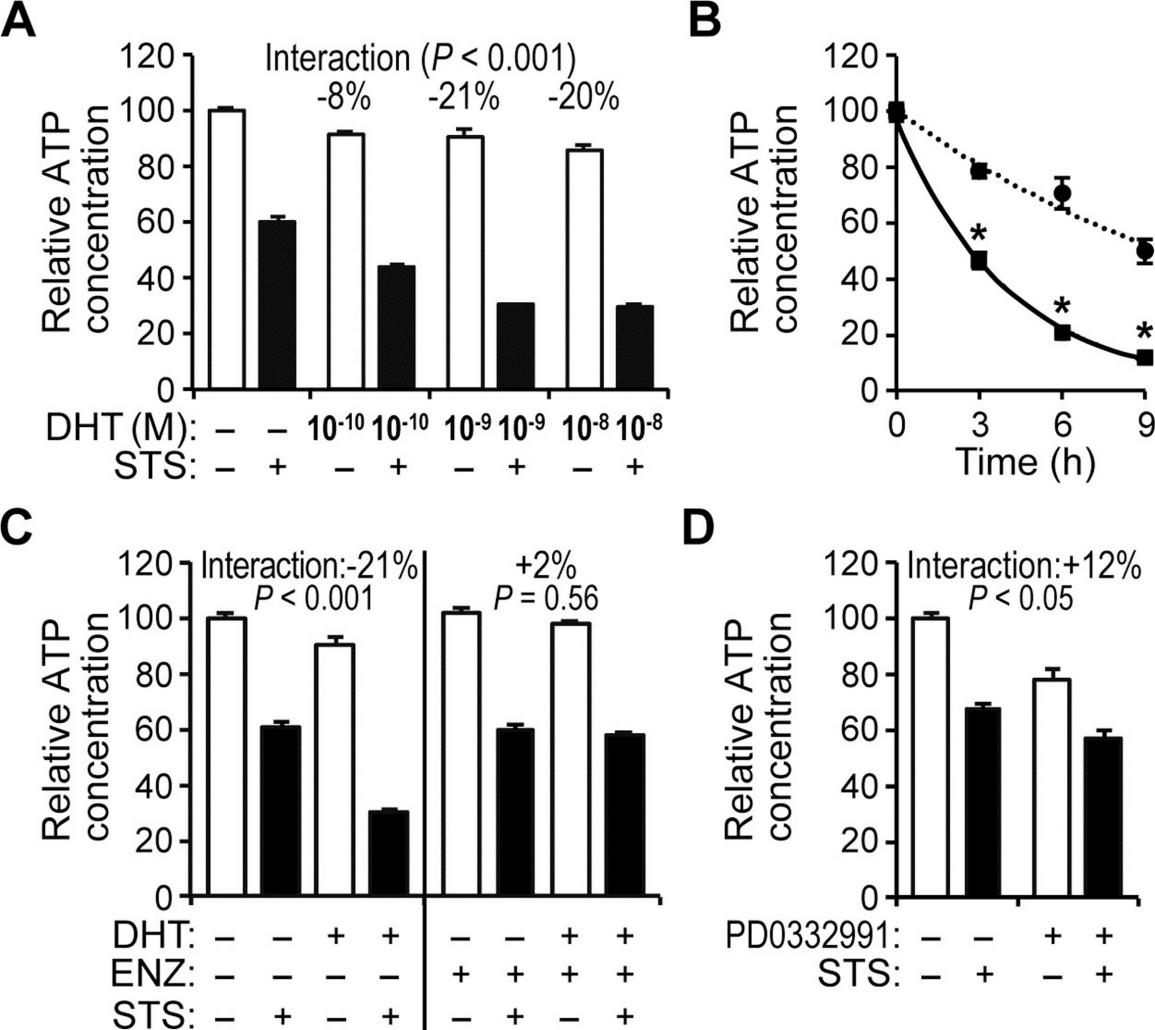
Androgen and staurosporine synergize to decrease the relative ATP concentration in HPr-1AR cells. (A) HPr-1AR cells were treated with a range of DHT concentrations or vehicle control for 18 hours and then co-treated with 1 μM STS or vehicle control for 6 hours. Relative ATP concentrations available for biochemical processes in metabolically active cells were quantified using a luciferase-based bioassay for relative ATP levels in cultured cells. In comparison to vehicle control, STS and to a lesser extent DHT significantly decrease the relative ATP concentration of HPr-1AR cells at 24 hours. In addition, ANOVA revealed significant interaction between 1 μM STS and 0.1–10 nM DHT, which is visually evident from the unparallel trends of the white bars and black bars in the plot. Estimates of the interaction effect and corresponding p-values are indicated. Negative interaction terms indicate synergy whereas positive values indicate antagonism between DHT and STS. (B) Cells were treated with 10 nM DHT or vehicle control for 24 hours and then co-treated with 0.5 μM STS for 0, 3, 6, or 9 hours (h). In comparison to control-treated HPr1AR cells (circles), DHT-treated HPr-1AR cells (squares) had 40%, 72%, and 76% reductions in ATP levels after 3, 6, and 9 hours of STS co-treatment, respectively. For time course analysis, significance differences between androgen treatment and vehicle control were determined at each time point using Student’s t-test and adjusted using the Bonferroni method, * P < 0.05. (C) Cells were treated with 1 nM DHT or vehicle control in the absence or presence of 5 μM enzalutamide (ENZ) for 18 hours and then co-treated with 1 μM STS or vehicle control for 6 hours. AR antagonist, ENZ significantly suppresses the synergistic interaction between DHT and STS. (D) Cells were treated with 5 μM PD0332991, a selective inhibitor of CDK4/6 kinase activity, for 18 hours to mimic the inhibitory effect of DHT on HPr-1AR cell cycle progression and growth, and then these cells were co-treated with 1 μM STS or vehicle control for 6 hours. The positive interaction term indicates that the synergy between DHT and STS on ATP depletion is not dependent on growth suppression and suggests an antagonistic effect between STS and PD0332991. Data represent the mean ± SEM (n ≥ 4).

There are a number of errors in the caption for Fig 2, “Androgen sensitizes HPr-1AR and RWPE-AR to apoptotic cell death.” Please see the complete, correct Fig 2 caption here.

**Fig 2:**
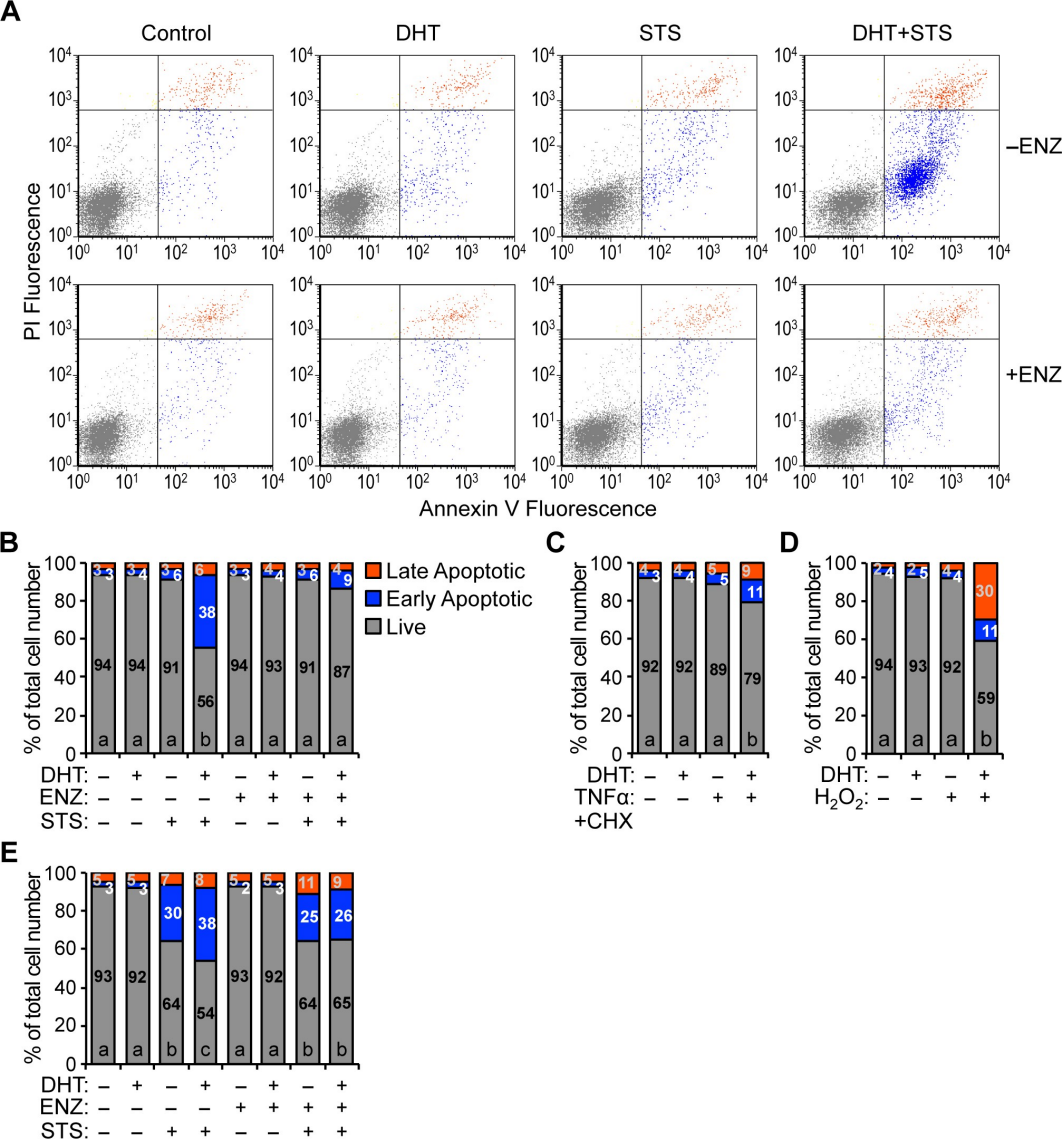
Androgen sensitizes HPr-1AR and RWPE-AR to apoptotic cell death. (A) HPr-1AR cells were treated with 1 nM DHT or vehicle control in the absence or presence of 5 μM ENZ for 19 hours and then co-treated with 1 μM STS or vehicle control for 5 hours. Cells were harvested, stained with annexin V and PI, and the fluorescence intensities of annexin V and PI stained cells were quantified by flow cytometry. Viable live cells (annexin V-negative and PI-negative, gray dots), early apoptotic cells (annexin V-positive and PI-negative, blue dots), and late apoptotic cells (annexin V-positive and PI-positive, orange dots) are indicated. (B) Quantification of the fraction of viable live (gray bar with black number), early apoptotic (blue bar with white number), and late apoptotic cells (orange bar with gray number) is shown from the dot plots in Fig 2A. DHT treatment alone does not trigger cell death in HPr-1AR. However, DHT sensitizes HPr-1AR to STS-induced apoptosis. In addition, AR antagonist, ENZ, suppresses the synergistic interaction between DHT and STS, which significantly increases the live cell proportion. (C) HPr-1AR cells were treated with 1 nM DHT or vehicle control for 12 hours and then co-treated with apoptosis inducer, TNFα+CHX, or vehicle control for 11 hours. The fluorescence intensities of annexin V and PI stained cells were then quantified by flow cytometry. DHT sensitizes HPr-1AR cells to apoptotic death induced by TNFα+CHX. (D) HPr-1AR cells were treated with 1 nM DHT or vehicle control for 20 hours and then co-treated with apoptosis inducer, H_2_O_2_, or vehicle control for 24 hours. The fluorescence intensities of annexin V and PI stained cells were then quantified by flow cytometry. DHT sensitizes HPr-1AR cells to apoptotic death induced by H_2_O_2_. (E) RWPE-AR cells were treated with 1 nM DHT or vehicle control in the absence or presence of 5 μM ENZ for 30 hours and then co-treated with 1 μM STS or vehicle control for 10 hours. The fluorescence intensities of annexin V and PI stained cells were then quantified by flow cytometry. DHT treatment alone does not induce cell death in RWPE-AR. However, DHT sensitizes RWPE-AR to STS-induced apoptosis. Further, ENZ co-treatment completely suppresses the synergistic interaction between DHT and STS, fully rescuing the live cell proportion of RWPE-AR. Data represent the mean (n ≥ 3). Comparisons between multiple treatment groups were performed using three- or two-way ANOVA followed by Tukey's honest significant difference test (S2 Table).

In the “Androgen sensitizes HPr-1AR and RWPE-AR to apoptotic cell death” subsection of the Results, there is an error in the first sentence of the second paragraph. The correct sentence is: To test whether the DHT-sensitized cell death is limited to STS-induced apoptosis, we treated HPr-1AR with other cell stress agents, including tumor necrosis factor-alpha and cycloheximide (TNFα+CHX), hydrogen peroxide (H_2_O_2_), and AT101, which can induce apoptosis through different mechanisms that are distinct from STS [55–59].

In the “Androgen sensitizes HPr-1AR and RWPE-AR to apoptotic cell death” subsection of the Results, there is an error in the third sentence of the second paragraph. The correct sentence is: DHT co-treatment potentiated TNFα+CHX-induced apoptosis by 10% (Fig 2C and S2 Table) and H_2_O_2_-induced cell death by 33% (Fig 2D and S2 Table).

There are a number of errors in the caption for Fig 3, “Androgen-sensitized apoptosis of HPr-1AR cells involves caspase activation.” Please see the complete, correct Fig 3 caption here.

**Fig 3:**
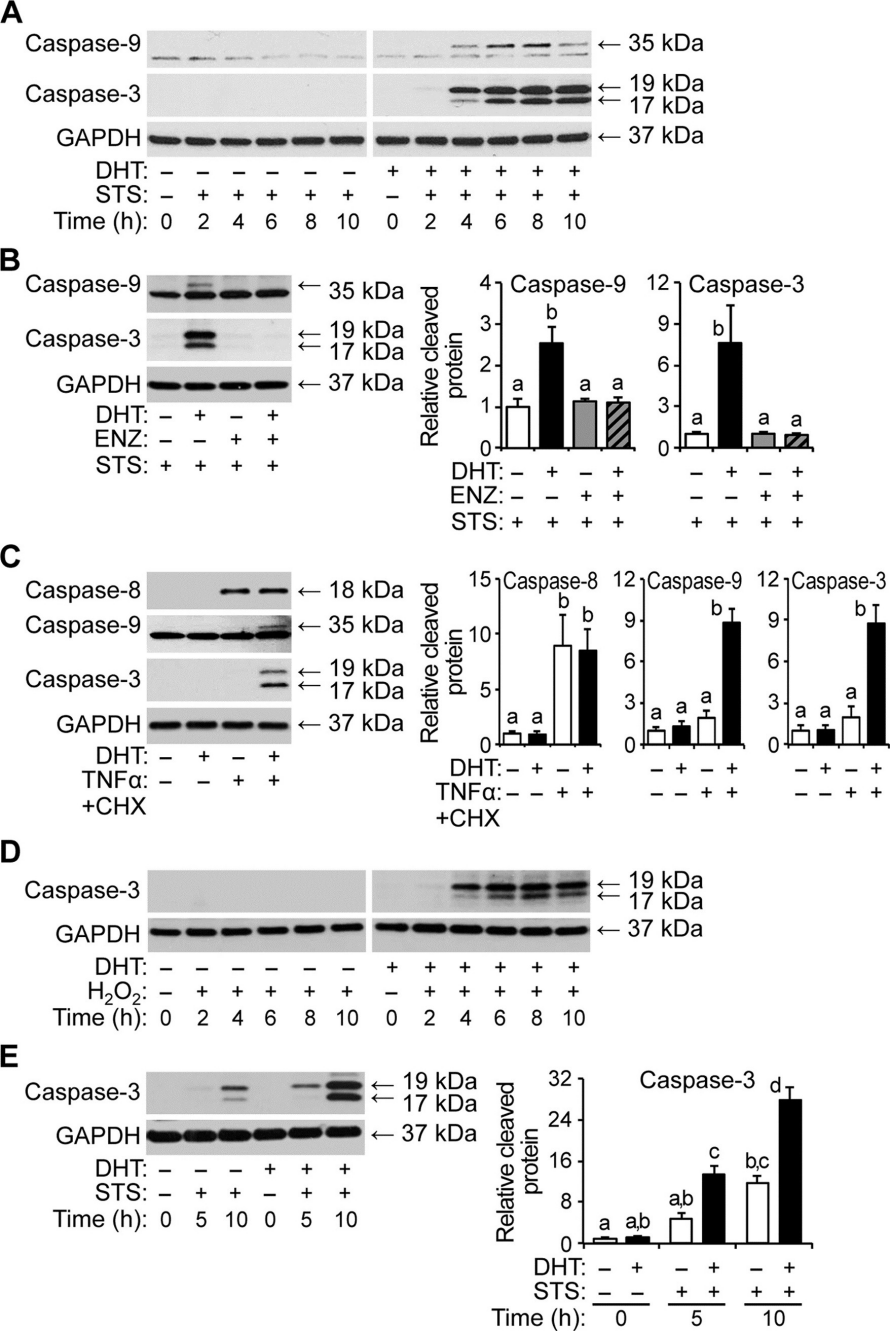
Androgen-sensitized apoptosis of HPr-1AR and RWPE-AR cells involves caspase activation. (A) HPr-1AR and RWPE-AR cells were treated with 10 nM DHT or vehicle control for 18 hours and co-treated with 1 μM STS for 0 to 10 hours. Immunoblot analysis was performed using antibodies that detect the cleaved and active forms of caspase-9 (35 kDa) and caspase-3 (19 and 17 kDa). HPr-1AR cells pretreated with DHT show rapid activation of caspase-9 and caspase-3 upon STS co-treatment, whereas DHT or STS treatment alone show little or no caspase activation. (B) Cells were treated with 1 nM DHT or vehicle control in the absence or presence of 5–10 μM ENZ for 18 hours and then co-treated with 1 μM STS or vehicle control for 6 hours. The DHTinduced cleavage of caspase-9 and caspase-3 in STS-treated HPr-1AR cells is completely suppressed by AR antagonist, ENZ. (C) Cells were treated with 1–10 nM DHT or vehicle control for 18 hours and then cotreated with TNFα+CHX or vehicle control for 10 hours. Immunoblot analysis was performed using an additional antibody to detect the cleaved and active form of caspase-8 (18 kDa), an initiator caspase that is activated in response to extrinsic apoptotic stimuli, such as TNFα. DHT and TNFα+CHX synergistically enhance cleavage of caspase-9 and caspase-3, whereas DHT or TNFα+CHX treatment alone shows no significant activation of caspase-9 or caspase-3. The arrows and corresponding molecular weights indicate the different caspase forms. (D) HPr-1AR cells were treated with 1 nM DHT or vehicle control for 24 hours and co-treated with 200 μM H_2_O_2_ for 0 to 10 hours. HPr-1AR cells pretreated with DHT show rapid activation of caspase-3 upon H_2_O_2_ co-treatment, whereas DHT or H_2_O_2_ alone show little or no caspase-3 activation. (E) RWPE-AR cells were treated with 1 nM DHT or vehicle control for 38 hours and then co-treated with 1 μM STS or vehicle control for 5 to 10 hours. RWPE-AR cells pretreated with DHT show rapid and robust activation of caspase-3 upon STS co-treatment (11-fold at 5 hours and 23-fold at 10 hours) compared to RWPE-AR cells pretreated with vehicle as a control. Immunoblot results were quantified and represented as the mean ± SEM (n ≥ 3). Comparisons between different treatments were performed using two-way ANOVA followed by Tukey's honest significant difference test.

There is an error in the penultimate sentence of the ninth paragraph of the Results section. The correct sentence is: Further, inhibition of transcription by DRB or protein synthesis by CHX, robustly suppressed the synergy between DHT and STS in HPr-1AR (Fig 5C).

There is an error in the caption for Fig 5, “Transcription and protein synthesis are necessary for androgen-sensitized apoptosis of HPr-1AR.” Please see the complete, correct Fig 5 caption here.

**Fig 5:**
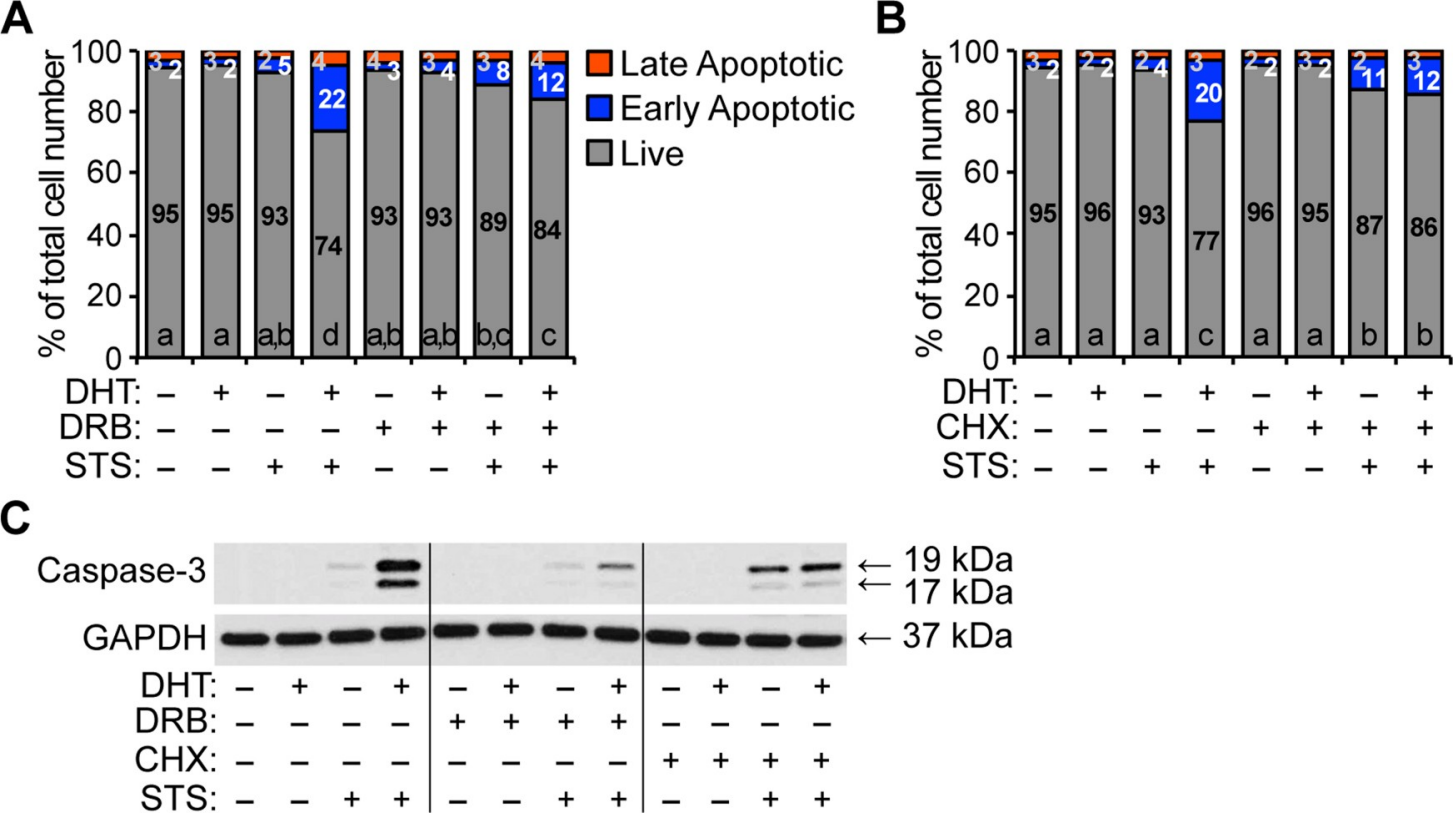
Transcription and protein synthesis are necessary for androgen-sensitized apoptosis of HPr-1AR. Cells were treated with 1 nM DHT or vehicle control in the absence or presence of transcription inhibitor, 20 μg/mL 5,6-dichlororibofuranosylbenzimidazole (DRB), for 16 hours, and then these cells were co-treated with 1 μM STS or vehicle control for 4 hours to induce apoptosis. Cells were harvested, stained with annexin V and PI, and the intensities of annexin V and PI stained cells were quantified by flow cytometry. DRB treatment significantly suppressed the androgen-sensitized apoptosis of HPr-1AR. (B) HPr-1AR cells were treated with 1 nM DHT or vehicle control in the absence or presence of protein synthesis inhibitor, 25 μg/mL CHX, for 16 hours, and then these cells were co-treated with 1 μM STS or vehicle control for 4 hours to induce apoptosis. Cells were harvested, stained with annexin V and PI, and analyzed by flow cytometry. CHX co-treatment completely suppressed the androgen-sensitized apoptosis of HPr1AR. Data represent the mean (n = 3). Comparisons between multiple treatment groups were performed using threeway ANOVA followed by Tukey's honest significant difference test (S2 Table). (C) Immunoblot analysis of cell lysates reveals that DHT-induced caspase-3 cleavage in STS-treated HPr-1AR cells is significantly suppressed by the inhibition of transcription (DRB) and protein synthesis (CHX).

In the “AR-mediated transcriptional regulation of apoptotic genes in HPr-1AR” subsection of the Results, there is an error in the fourth sentence of the first paragraph. The correct sentence is: In addition, transcripts for the AIFM2 gene, which codes for a pro-apoptotic protein that is released from the mitochondria into the cytoplasm upon MOMP, and the APAF1 gene, which codes for an apoptosis initiator protein that binds cytochrome *c* and forms the oligomeric apoptosome, were DHT-induced.

In the “Apoptotic functions of AR signaling” subsection of the Discussion, there is an error in the seventh sentence of the first paragraph. The correct sentence is: STS, H_2_O_2_, and AT101 stimulate the intrinsic apoptotic pathway by non-selective inhibition of protein kinases [54], oxidative stress and damage [57–59], and suppression of pro-survival BCL2 family genes [55], respectively; whereas, TNFα stimulates the extrinsic apoptotic pathway by activation of TNF family death receptors located at the cell surface [56].

In the “Apoptotic functions of AR signaling” subsection of the Discussion, there is an error in the fourth sentence of the second paragraph. The correct sentence is: Meanwhile, the activation of caspase-9, a hallmark of intrinsic apoptotic signaling, was detected after co-treatment with DHT and TNFα+CHX (Fig 3).
