# Supplementary figures and images for: Androgen-Sensitized Apoptosis of HPr-1AR Human Prostate Epithelial Cells
Source: PLoS One. 2016 May 20;11(5):e0156145. doi: 10.1371/journal.pone.0156145 (PMC4874596; doi:10.1371/journal.pone.0156145)

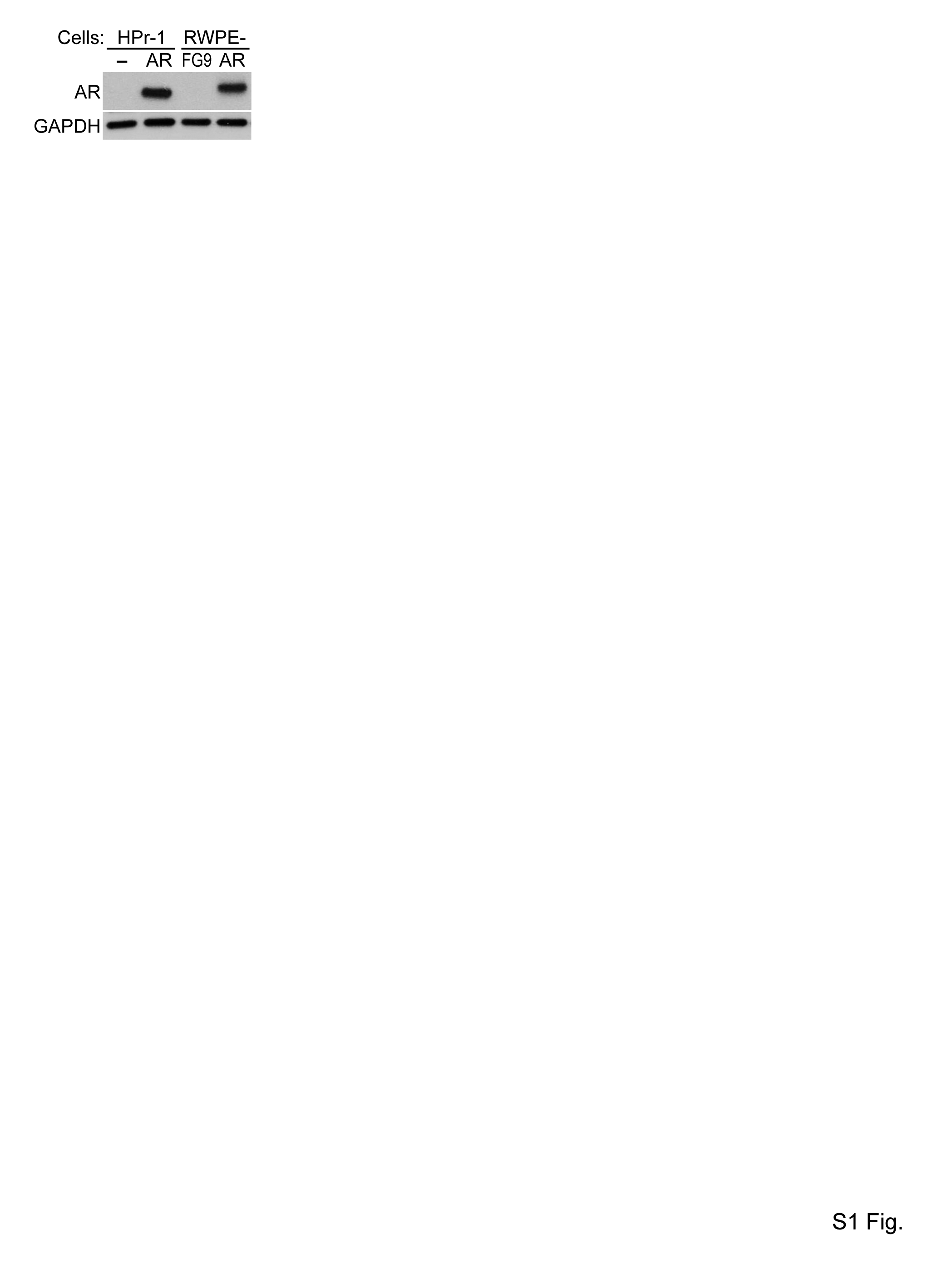

Supplement: S1 Fig — Immunoblots show robust AR protein expression in HPr-1AR and RWPE-AR lysates compared to HPr-1 and RWPE-FG9 lysates. AR protein expression is nearly 3-fold higher in HPr-1AR compared to RWPE-AR. (TIF) [file pone.0156145.s001.tif]

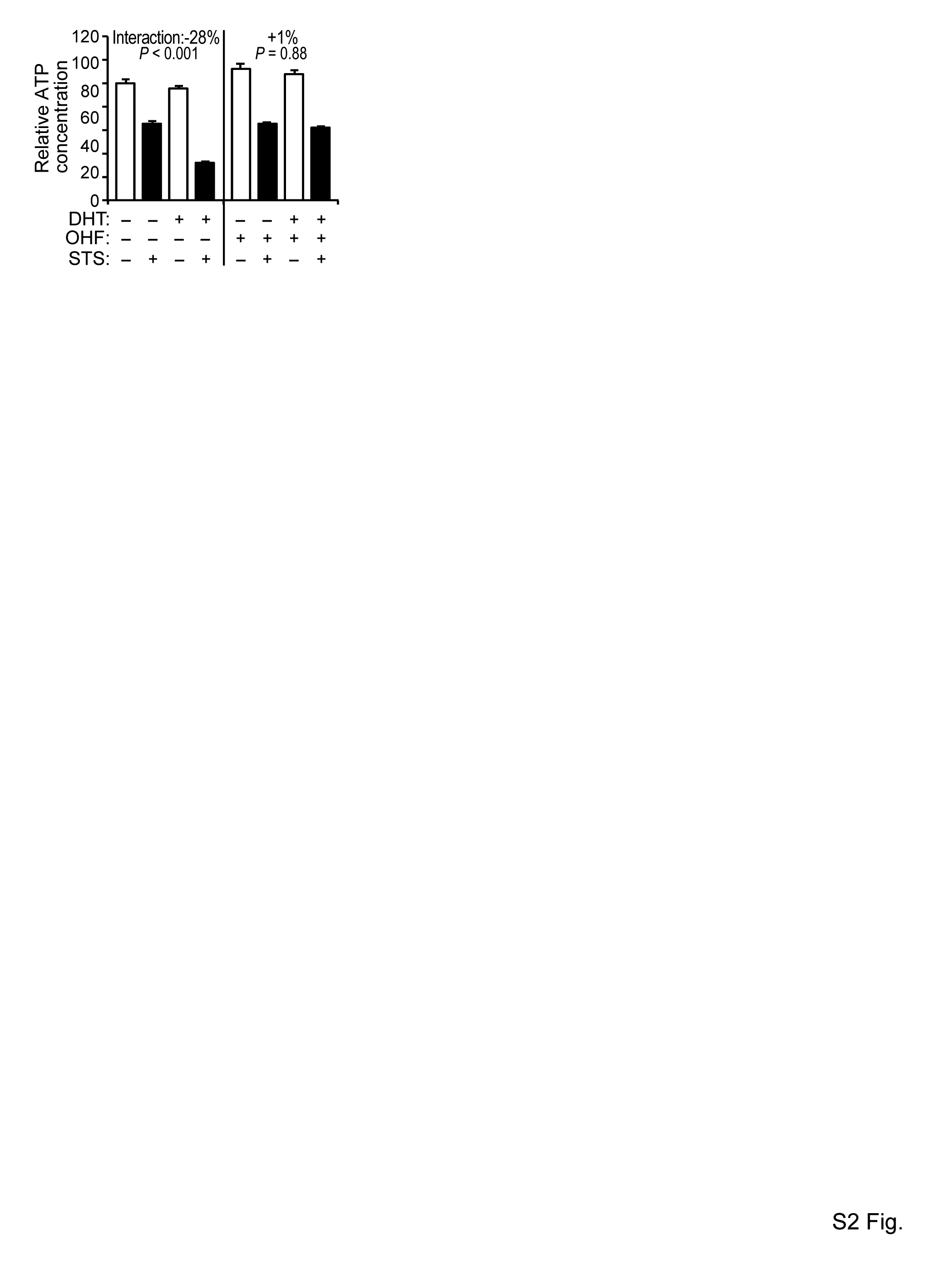

Supplement: S2 Fig — Cells were treated with 1 nM DHT or vehicle control and 10 μM 2-hydroxyflutamide (OHF) for 18 hours and then co-treated with 1 μM STS or vehicle control for 6 hours. AR antagonist, OHF, significantly suppresses the synergistic interaction between DHT and STS. Data represent the mean ± SEM (n = 4). (TIF) [file pone.0156145.s002.tif]

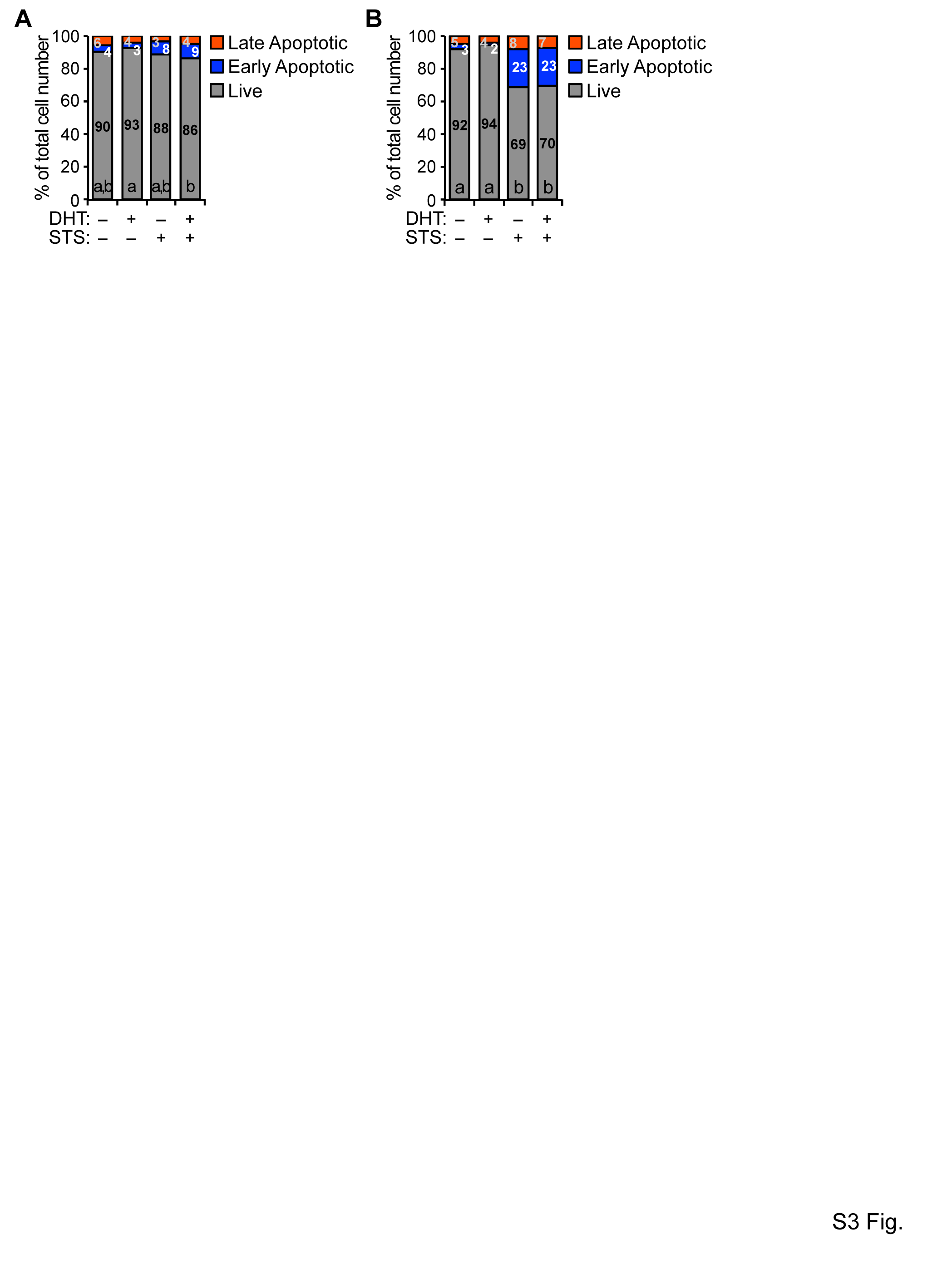

Supplement: S3 Fig — (A) HPr-1 cells were treated with 1 nM DHT or vehicle control 21 hours and then co-treated with 0.5–1 μM STS or vehicle control for 4 hours. Cells were harvested, stained with annexin V and PI, and the fluorescence intensities of annexin V and PI stained cells were quantified by flow cytometry. Quantification of the fraction of viable live (gray bar with black number), early apoptotic (blue bar with white number), and late apoptotic cells (orange bar with gray number) is shown. DHT treatment alone does not trigger cell death in HPr-1. Further, DHT does not sensitize HPr-1 to STS-induced apoptosis. (B) RWPE-FG9 cells were treated with 1–10 nM DHT or vehicle control for 29 hours and then co-treated with 1 μM STS or vehicle control for 10 hours. The fluorescence intensities of annexin V and PI stained cells were then quantified by flow cytometry. DHT treatment alone does not induce cell death in RWPE-FG9. Further, DHT does not sensitize RWPE-FG9 to STS-induced apoptosis. Data represent the mean (n = 3). Comparisons between multiple treatment groups were performed using two-way ANOVA followed by Tukey's honest significant difference test (S2 Table). (TIF) [file pone.0156145.s003.tif]

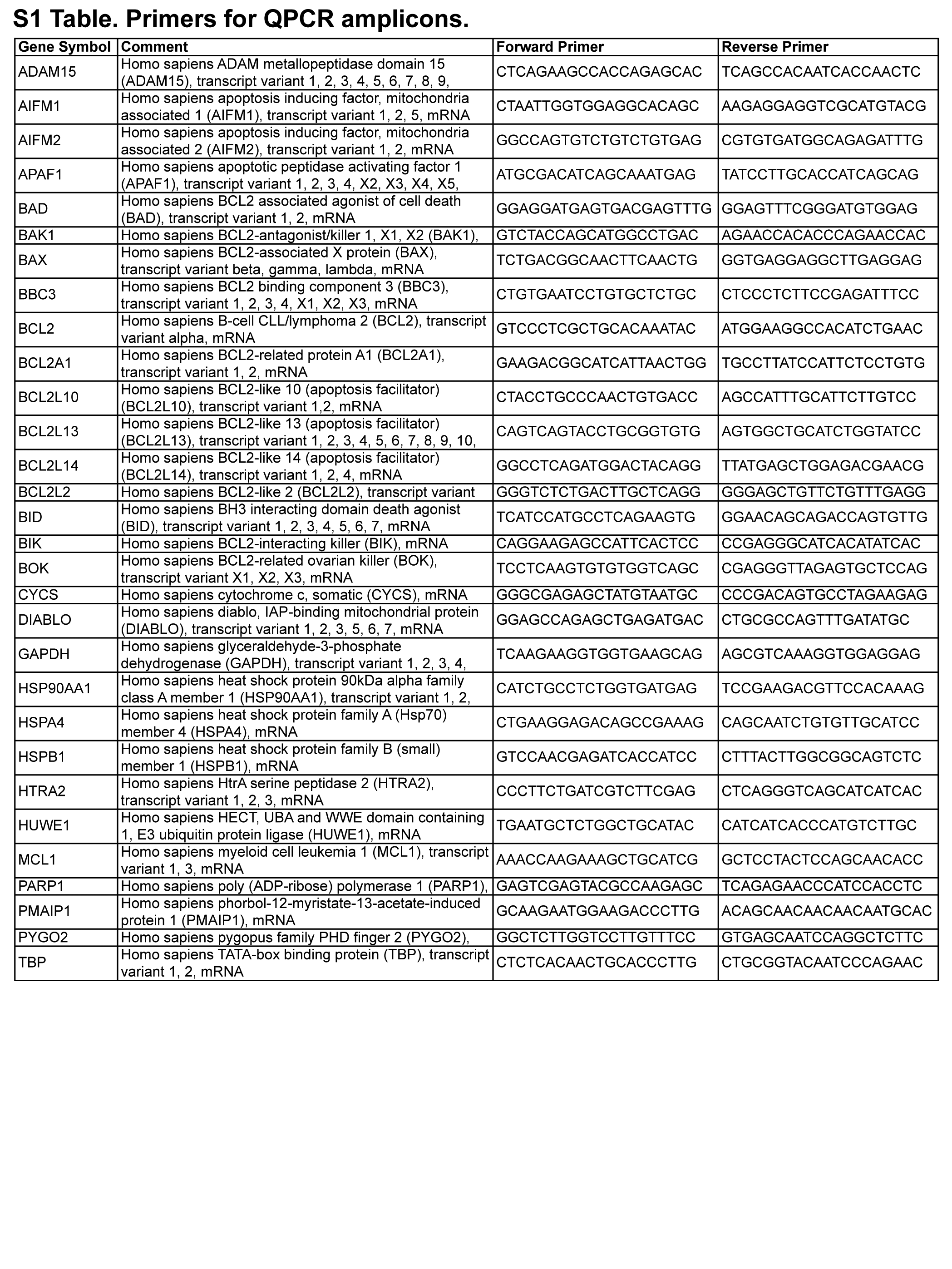

Supplement: S1 Table — (TIF) [file pone.0156145.s004.tif]

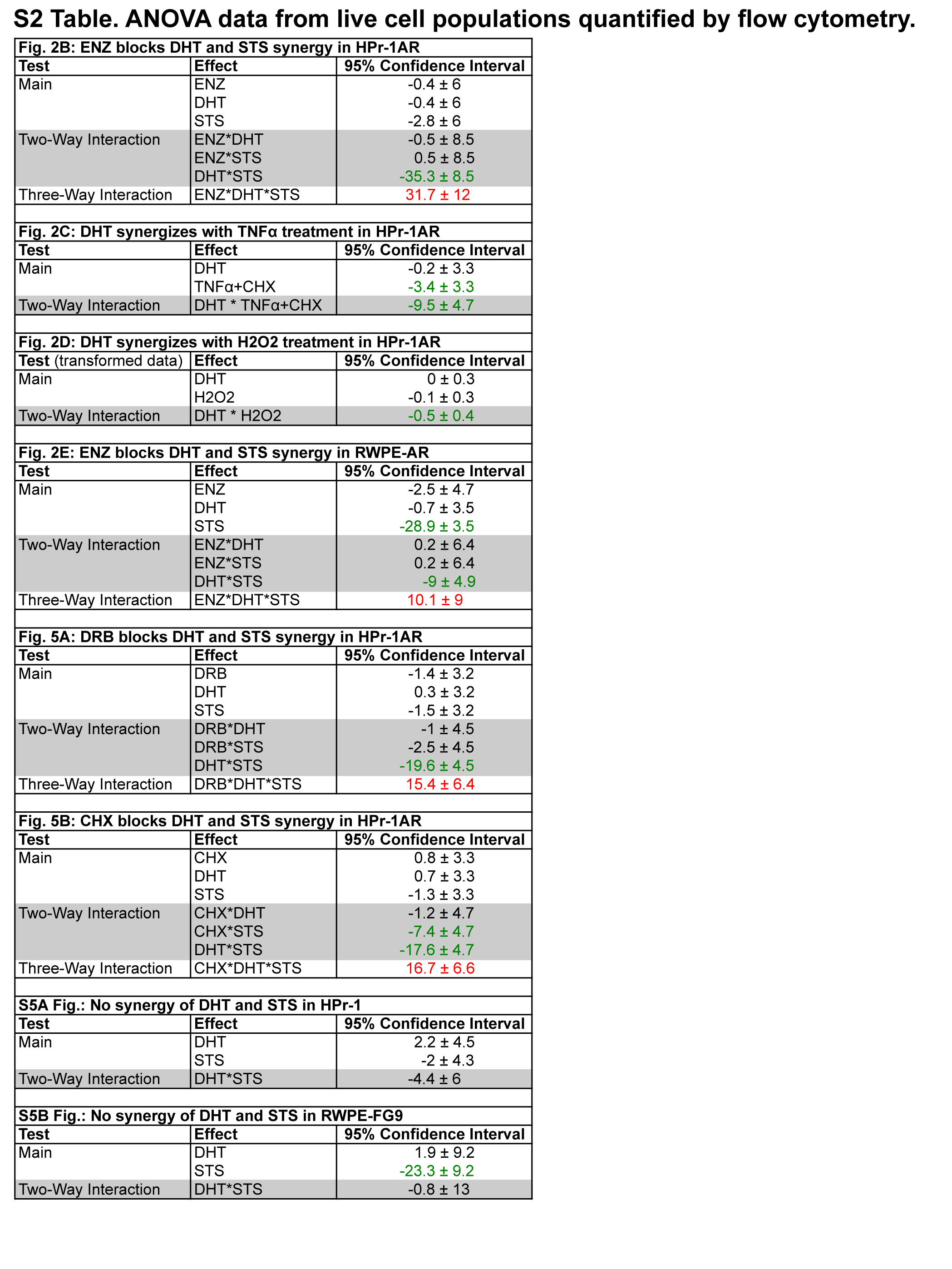

Supplement: S2 Table — (TIF) [file pone.0156145.s005.tif]
